# Research on the impact of artificial intelligence on the export technological complexity of chinese manufacturing enterprises: An analysis based on mediating effects

**DOI:** 10.1371/journal.pone.0351061

**Published:** 2026-07-02

**Authors:** Haiying Lin, Mengtian Wang, Meng Guo, Zhijuan Wang, Ziyuan Cheng, Wenlong Li, Muhammad Umer Arshad

**Affiliations:** 1 School of Business, Inner Mongolia University of Finance and Economics, Hohhot, Inner Mongolia, China; 2 School of Resource and Environmental Economics, Inner Mongolia University of Finance and Economics, Hohhot, Inner Mongolia, China; 3 Inner Mongolia Honder College of arts and Science, Hohhot, Inner Mongolia, China; 4 Department of Crop Sciences, University of Illinois at Urbana‐Champaign, Urbana, Illinois, United States of America; Industrial University of Ho Chi Minh City, VIET NAM

## Abstract

Technological innovation drives high-quality economic development, and artificial intelligence (AI) represents a new impetus for developing productive forces with new qualities. AI is becoming a focal point in economic development plans and national strategies worldwide due to its contribution to economic growth and the transformation of traditional production methods. This paper examines the impact and mechanism of AI on the export technological complexity of Chinese manufacturing enterprises from a corporate perspective. It utilizes data from listed manufacturing companies on the Shanghai and Shenzhen A-shares from 2008 to 2021 and employs a fixed-effects model. The results indicate that: (1) AI positively promotes the export technological complexity of Chinese manufacturing enterprises, with more pronounced effects in regions with higher export technological complexity. (2) Heterogeneity analysis indicates that AI significantly enhances the export technological complexity across various categories of enterprises. Particularly notable impacts are observed among state-owned enterprises, light textile enterprises, and enterprises located in the eastern and central regions. (3) Mechanism analysis reveals that AI indirectly promotes the export technological complexity of manufacturing enterprises by improving labor structure and enhancing corporate innovation capabilities. This study proposes relevant policy recommendations from four aspects: strengthening AI technology research and application, optimizing labor structure, enhancing corporate innovation development, and promoting balanced AI development.

## 1. Introduction

With the rapid advancement of technology, artificial intelligence (AI) has deeply permeated various industries, becoming a key driver of societal development. China’s export technological complexity in manufacturing has garnered widespread attention. The report from the 20th National Congress of the Communist Party of China states: “Relying on the advantages of China’s super-large-scale market, we should attract global resources through domestic circulation, enhance the linkage effect of both domestic and international markets and resources, and improve the quality and level of trade and investment cooperation.” According to Hausmann et al. (2007), export technological complexity reveals the structure and quality of a country’s export products and reflects a country’s resource endowments, trade layout, and production technology levels [1]. Dai Xiang and Zheng Lan (2015) believe enhancing export technological complexity can drive quantitative economic growth and achieve qualitative leaps [2]. In recent years, AI, as a frontier technology with high penetration and application, has increasingly highlighted its status in the global economy. Goldfarb and Trefler (2018) showed that smart technology empowers export enterprises, opening new paths for their growth [3]. China’s industrial robot installations soared to 243,300 units in 2021, accounting for half of the global total, with a growth rate of 44% (Data Source:International Federation of Robotics. https://ifr.org/). Despite the large usage of industrial robots in China, most are still imported, with imports exceeding 70%. China’s industrial robot imports reached USD 2.196 billion in 2021, with multifunctional industrial robots making up the highest proportion. It is estimated that by 2026, the core industry of AI will exceed 600 billion RMB, with its application scenarios expanding from traditional consumer and internet fields to more industries such as manufacturing, energy, and power (Data Source: China Custom, http://www.customs.gov.cn/). In the current context of continuous innovation in digital and intelligent technologies, new technologies, production methods, and products such as big data and AI are emerging, injecting new vitality into economic growth and developing enterprise export trade.

Aghion et al. (2017) particularly point out that even in highly automated contexts, the capital share and economic growth rate may stabilize rather than increase indefinitely. In recent years, research has further deepened to the level of micro-level mechanisms [4]. Building upon this theoretical framework,a substantial body of research has focused on the specific effects of AI on the labor market.Most research on the economic impact of robots and AI originates from developed Western countries and mainly focuses on labor markets and economic growth. Regarding the impact of AI on the labor market, Gregory et al. believe that using industrial intelligence can increase product demand and create new jobs, thereby promoting employment growth [5]. On the other hand, Acemoglu and Restrepo found that increasing the use of robots reduces employment in the U.S. labor market [6]. Dauth et al., using German IFR data, showed that robots did not cause overall job losses in Germany but changed the employment structure, indicating that robots and AI have varying impacts on different industries [7]. Koch et al. (2021) found that the application of robots significantly promotes the upskilling of employment structures within enterprises, while simultaneously enhancing firm productivity and export propensity. This provides direct evidence for understanding the micro-level impacts of artificial intelligence [8].In terms of economic growth, Chen Yanbin et al. constructed a dynamic general equilibrium model, showing that AI has the potential to mitigate the economic impact of aging and thus promote economic growth [9]. Similarly, Kromann et al. pointed out that robots and industrial intelligence positively affect economic growth and can improve total factor productivity [10].Yang Guang and Hou Yu further supported these views, revealing the different impacts of demographic dividends on economic growth at various stages [11]. The “AI productivity paradox” highlighted by Brynjolfsson et al. suggests that the realization of its positive impacts may depend on complementary investments in areas such as organizational structure and skills. This implies that the effects may exhibit significant heterogeneity [12].

Although the economic impact of artificial intelligence has been widely discussed, several research directions remain to be further explored. On the one hand, most studies focus on macro-level economic growth and the labor market. Empirical evidence systematically examining the impact of AI on export technology sophistication from a micro-level firm perspective remains relatively limited. On the other hand, research on its underlying mechanisms, particularly empirical tests based on mediating mechanisms such as the optimization of firm labor structure and the enhancement of innovation capability, is still insufficient.

Based on the aforementioned research gaps, the marginal contributions of this study are as follows: First, in terms of research perspective, it shifts the analysis from the macro-aggregate level down to the micro-firm level. Using data from Chinese listed companies, it directly examines the impact of AI on firms’ export technology sophistication, providing new evidence for understanding the micro-level mechanisms of AI. Second, in terms of research content, it not only verifies the direct impact of AI but further employs a mediating effect model to empirically reveal two key pathways: “optimizing labor structure” and “enhancing firm innovation capability,” thereby deepening the understanding of AI’s influence mechanisms. Third, in terms of analytical dimensions, it conducts heterogeneity analyses from multiple perspectives, including firm ownership, industry technological attributes, and regional geographical location, revealing the differential effects of AI and making the research conclusions more policy-relevant.

Upon reviewing the existing literature, some areas still require further exploration: (1) Since AI technology innovation is still in its early stages, relevant statistical data is difficult to obtain. Most studies rely on industrial robots as a single indicator or limited digital statistics to evaluate AI levels. This leads to an incomplete indicator system that fails to reflect the actual level of AI technology innovation fully. Therefore, the AI indicator evaluation system needs further optimization and improvement. (2) Empirical research primarily focuses on AI and manufacturing panel data from more than ten developed countries. At the same time, there is relatively less research using meso-level data from enterprises to study the impact of AI on manufacturing exports. Considering that enterprises are the main bodies of innovation and production, it is indispensable to study the impact of AI on manufacturing exports from a meso-level perspective. Thus, future research should focus more on the corporate level to deeply explore the effects and mechanisms of AI on manufacturing exports.

This paper focuses on two aspects: (1) export behavior at the corporate level, using data from listed companies in the Cathay Pacific and other databases to deeply analyze the impact of AI on the export technological complexity of enterprises, aiming to provide new empirical evidence for understanding the influence of AI on the export technological complexity of manufacturing enterprises. (2) Using data from listed manufacturing companies on the Shanghai and Shenzhen A-shares from 2008 to 2021 and employing a fixed-effects model, examines the impact of AI on the export technological complexity of Chinese manufacturing enterprises, further analyzing its heterogeneity and exploring its impact mechanism through mediating variables. This study expands the application scope and impact mechanism of AI in the economic field.

The innovative points of this paper are reflected in the following two aspects: 1. Research Perspective: This paper focuses on the export behavior of micro-enterprises, utilizing data from listed companies obtained from databases such as CSMAR. From a micro-enterprise level, it conducts an in-depth exploration of the impact of artificial intelligence (AI) on the export technological sophistication of enterprises. This shift in research perspective is expected to provide new empirical evidence for a comprehensive understanding of the influence of AI on the export technological sophistication of manufacturing enterprises. 2. Research Content: As an emerging research topic, the impact of AI on the economy has not yet received extensive academic attention. Although some studies have touched upon its effects on economic growth, employment and other related aspects, the literature exploring how AI affects the export technological sophistication of manufacturing enterprises remains relatively scarce. This paper attempts to establish a connection between AI and the export technological sophistication of manufacturing enterprises, which not only expands the application scope of AI in the economic field but also enriches the research content related to the export technological sophistication of manufacturing enterprises.

## 2. Theoretical analysis and research hypotheses

### 2.1. Theoretical analysis

Considering that artificial intelligence (AI) may influence export technological complexity through labor structure and corporate innovation capabilities, this study constructs a heterogeneous firm trade model that includes labor productivity and technological levels. Suppose there is an industrial sector within a monopolistic competition market, with N firms operating in this sector, producing differentiated products with varying levels of technology. Assume that the product p is continuous and falls within the range 0 ≤ p ≤ 1.1.

#### 2.1.1. Demand.

Assume the CES (Constant Elasticity of Substitution) consumer utility function can be expressed as:


$$\begin{array}{c} U=(\int_{\text{0}}^{\text{1}}t(p{)}^{\text{1}-\rho }c(p{)}^{\rho }dp{)}^{\frac{\text{1}}{\rho }},\;\text{0}<\rho <\text{1} \end{array}$$ (1)

Where p is one type of the total consumption product P, t(p) represents the technological innovation capability contained in the product, and c(p) denotes the consumption quantity of the product. The parameter ρ=(σ−1)/σ, where σ represents the elasticity of substitution. When σ→∞, implying ρ = 1, the products are homogeneous and nearly perfectly substitutable, resulting in non-differentiated consumption of any product. When σ < 1, implying ρ < 0, the products are complementary and differentiated.

At this point, consumers have the following budget constraints:


$$\begin{array}{c} max\int_{\text{0}}^{\text{1}}n(p)c(p)dp=y \end{array}$$ (2)

Where n(p) represents the product price and c(p) denotes the product consumption quantity. When utility is maximized, combining Equations (1) and (2), the optimal demand for product p can be calculated as:


$$\begin{array}{c} (z)=\frac{t(z)n(z{)}^{-\sigma }R}{N^{\text{1}-\sigma }} \end{array}$$ (3)

where total income is 𝑅, defined as


$$\begin{array}{c} \;R=\int_{\text{0}}^{\text{1}}n(p_{i})c(p_{i})dp_{i}; \end{array}$$

The aggregate price index is:


$$\begin{array}{c} P={\left[\int_{\text{0}}^{\text{1}}n(p_{i}{)}^{\text{1}-\sigma }t(p_{i})dp_{i}\right]}^{\frac{\text{1}}{\text{1}-\sigma }} \end{array}$$

Considering the technological content of products. From the function of P, it is evident that P is endogenous, but for a single firm, it can be considered exogenous. This is because the presence of N firms in the industry means that the scale of a single firm cannot affect the aggregate price index.

#### 2.1.2. Supply.

Assume each new entrant in the industry chooses to produce a differentiated product and has monopolistic pricing power. In a monopolistic competition market, each market is open and there are no monopoly profits. Suppose that in this industry’s supply market, the N firms producing differentiated products incur both variable and fixed costs. The economies of scale characteristics of the firms can be realized through the setting of fixed costs. If firms choose to export, additional export costs will be incurred. In a monopolistic competition market, each market is open and there are no monopoly profits (Hallak and Sivadasan, 2009) [13]. According to the heterogeneous firm trade model, assume the export cost follows an iceberg cost structure, and firms’ heterogeneity is reflected in labor structure 𝑙(p) and innovation capability t(p). The changes in these two aspects affect costs. Referring to the heterogeneous firm trade model by Melitz and Redding (Melitz and Redding, 2014) [14]:


$$\begin{array}{c} MC[\varphi ,t(\varphi )]=\frac{t(\varphi {)}^{\beta }}{\varphi },F(t(\varphi ))=f+ft(\varphi {)}^{\gamma } \end{array}$$ (4)

Where MC is marginal cost, F is fixed cost, φ is the firm’s labor structure, t(φ) is the technological innovation content of the product, β is the technology elasticity of marginal cost (β > 0), and f represents exogenous fixed costs, with γ being the technology elasticity of endogenous fixed costs (γ > 0).

#### 2.1.3. Market equilibrium.

According to the Dixit-Stiglitz model (Dixit and Stiglitz, 1977) [15], we find that for market equilibrium, two conditions must be met. First, firms need to satisfy the profit maximization condition in Equation (3). Second, because the market is fully open, new firms will continually enter as long as there are profits, until there are no profits in the entire market. Therefore, in equilibrium, total firm profits are zero. Since each new entrant in the industry chooses to produce a differentiated product with monopolistic pricing power, firms will adopt monopolistic pricing, leading to the following profit maximization problem:


$$\begin{array}{c} max\pi =n(\varphi )x-[f+ft(\varphi {)}^{\gamma }+\frac{t(\varphi {)}^{\beta }}{\varphi }]x \end{array}$$ (5)

Where x refers to the quantity of product sales. Assuming export costs follow an iceberg model, the price n of the products produced by each firm in the market does not affect the price index N. Thus, firms can adopt a markup pricing approach, resulting in the following equilibrium price:


$$\begin{array}{c} N^{*}[\varphi ,t(\varphi )]=\frac{\sigma }{\sigma -\text{1}}\frac{\lambda (\varphi {)}^{\beta }\tau }{\varphi } \end{array}$$ (6)

Combining Equations (6) and (5), we can derive the technological complexity of the export product:


$$\begin{array}{c} t(\varphi {)}^{r-\text{1}-\beta +\beta \sigma }=\frac{(\text{1}+\beta -\beta \sigma )R}{\sigma^{\sigma }f\gamma p^{\text{1}-\alpha }}{\left[\frac{(\sigma -\text{1})\varphi }{\tau }\right]}^{\sigma -\text{1}} \end{array}$$ (7)

Where 0<1+β−βσ<γ. Thus, we can observe that the export technological complexity of firms is positively proportional to labor structure and technological level.

### 2.2. Hypotheses

#### 2.2.1. Impact of artificial intelligence on the export technological complexity of manufacturing enterprises.

In the context of rapid economic and technological development, artificial intelligence (AI) is gradually becoming a key driver for transforming and upgrading China’s manufacturing industry. Its role in enhancing the export technological complexity of Chinese manufacturing enterprises cannot be underestimated. Yang Xiaoxia et al. (2024) argue that artificial intelligence has a significant promoting effect on the enhancement of export technological sophistication in China’s manufacturing industry [16]. Meanwhile, Chen Jia et al. (2023) also pointed out in their research that artificial intelligence plays a significant role in improving the export technological sophistication of the manufacturing sector [17]. Firstly, according to the theory of comparative advantage, each country should specialize in producing goods in which it has a relative advantage and export these goods in exchange for those in which it does not have an advantage [18]. Therefore, AI technology has given Chinese manufacturing a significant comparative advantage in technological innovation. Advanced technologies such as deep learning and big data analysis provide manufacturing enterprises with comprehensive support from product design to manufacturing, enabling Chinese manufacturers to understand market demands better and develop more competitive products [19]. This not only enhances the technological content of products but also improves the reputation and status of Chinese manufacturing in the international market, thereby increasing the export technological complexity.

Secondly, the theory of competitive advantage emphasizes that enterprises gain an edge over competitors through innovation and management optimization. In the manufacturing sector, AI significantly enhances production efficiency through the application of automation and intelligent production methods. The gradual accumulation of cost advantages provides enterprises with more funds for research and development, promoting continuous technological upgrades. This high-efficiency production method ensures high-quality products and timely delivery, giving Chinese manufacturing a valuable competitive edge in the international market.

Furthermore, AI has assisted the manufacturing sector in upgrading its industrial structure. As AI technology becomes more widespread and deeply integrated, the manufacturing industry is gradually transitioning from low-tech and low-value-added industries to high-tech and high-value-added industries. This transition significantly improves the overall technological level of Chinese manufacturing, as well as the technological content and added value of export products, thereby increasing export technological complexity. AI also enhances the international market’s brand influence of enterprises, further promoting the export technological complexity of Chinese manufacturing. Enterprises using AI technology to precisely target customers and provide high-quality, personalized products and services not only increase their visibility and reputation in the international market but also attract more high-end customers and partners, further enhancing export technological complexity. Therefore, this transformation of Chinese manufacturing is achieved by improving the technological content of export products and enhancing brand influence.

Based on the above discussion, this paper proposes the first hypothesis:

*Hypothesis H1: AI can enhance the export technological complexity of Chinese manufacturing enterprises.*

#### 2.2.2. Impact of AI on the export technological complexity of manufacturing enterprises by improving labor structure.

AI, as a new generation of technological progress, has significantly impacted the employment market in China. Research on this impact is mainly divided into two categories: some studies conclude that AI will reduce traditional job positions and generally have a negative effect on overall employment [20]; other studies find that AI stimulates enterprise development and the production of larger quantities of products or the establishment of new integration fields [21], which will inevitably bring more employment opportunities. Zhao Chunming et al. (2025) points out that the labor allocation optimization effect and technological innovation effect of smart manufacturing are important mechanisms influencing the enhancement of export technical sophistication [22]. AI technology can only replace some labor, while it cannot substitute labor that is difficult to automate and requires high skills [6]. Additionally, under AI technology, the labor structure will shift from operational and technical employees to knowledge-based employees, which will undoubtedly demand higher skills from the workforce [23]. According to Hémous and Olsen’s research, they also believe that the use of AI can reduce the need for low-skilled labor, but can increase labor productivity and increase the demand for high-skilled labor [24]. Moreover, industrial structural transformation and upgrading will lead to an increase in relevant skill positions, providing workers with more vocational training opportunities and thus expanding the demand for human capital in production departments [25]. Since the key factor for upgrading the export technological complexity of the manufacturing industry is the optimization of labor structure leading to high-quality employment, optimizing the labor structure helps achieve the upgrade of the export technological complexity of Chinese manufacturing enterprises.

Based on the above analysis, this paper proposes the second hypothesis:

Hypothesis H2: AI can improve the export technological complexity of Chinese manufacturing enterprises by enhancing the labor structure.

#### 2.2.3. Impact of AI on the export technological complexity of manufacturing enterprises by enhancing innovation capability.

As the core product of the fourth technological revolution, AI plays a crucial role in promoting regional innovation capabilities and injecting new vitality into regional development. Jin Zehu et al. (2023) indicates that artificial intelligence can not only directly promote the high-quality development of China’s manufacturing exports but also indirectly enhance the level of high-quality development in manufacturing exports by improving both production efficiency and innovation efficiency [26]. Regions are now developing innovation paths with local characteristics based on their resource features or national strategies. The introduction of AI provides strong innovation development momentum to these areas. This momentum is concentrated in three points:

Firstly, AI provides economic support for regional innovation development by reducing innovation costs. During production, intelligent labor can reduce reliance on large amounts of human labor and lower related costs. Its powerful data processing and analysis capabilities offer clear directions for optimizing resource allocation in R&D and enhancing the efficiency of innovation activities, enabling regions to grasp market trends more accurately.

Secondly, AI enhances the efficiency of regional innovation development through optimal resource allocation. Industries with high technological content, high productivity, and low costs expand under AI guidance, leading to the modernization of export technologies. AI can also guide innovation elements like manpower, capital, and technology to key sectors, reducing resource misallocation and improving innovation efficiency.

Thirdly, AI provides sustained momentum for regional development by promoting technological innovation. The creativity and technological nature of AI can bring about product innovation and innovations in technology, markets, and other areas, continuously injecting vitality into regional innovation development. The ongoing enhancement of AI’s creativity and technological aspects will continue to drive these advancements.

Innovation’s role in export technological complexity is widely recognized. Scholars generally believe that innovation effectively enhances export technological complexity by improving production efficiency and increasing the technological content of products. Additionally, technological spillovers and economies of scale among innovation entities and improvements in the innovation environment—such as the development of information technology and market competition levels—positively regulate export technological complexity. Therefore, innovation entities, innovation investment, and the innovation environment jointly form the key elements driving technological upgrades, creating an innovation closed-loop within the international division of labor system.This is also confirmed by the empirical study of Tian et al. (2021), who found that these three types of innovation factors have a significant role in promoting the technical complexity of exports [27].

Based on the above analysis, this paper proposes the third hypothesis:

*Hypothesis H3: AI can enhance the export technological complexity of Chinese manufacturing enterprises by boosting innovation.*

**Fig1:**
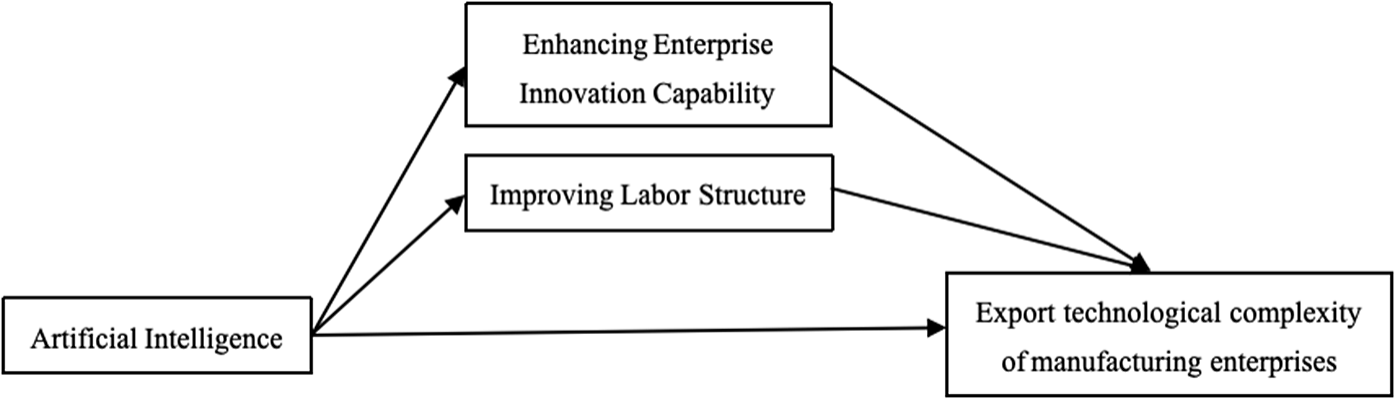
Mechanism Diagram of How Artificial Intelligence Affects the Export Technological Complexity of Enterprises by Promoting Regional Innovation Development.

The pathways through which AI affects the export technological complexity of manufacturing enterprises are shown in Fig 1.

## 3. Research design

### 3.1. Constructing the econometric model

We aim to examine the impact of artificial intelligence on the export technological complexity of Chinese manufacturing enterprises. Therefore, we constructed the following baseline econometric model for estimation:


$$\begin{array}{c} Ln\left(ESI_{ijt}\right)=\delta_{\text{0}}+\delta_{\text{1}}AI_{ijt}+\sum \delta icontrolit+\gamma_{j}+\gamma_{t}+\varepsilon_{ijt} \end{array}$$ (8)

The subscripts I, j, and t represent enterprises, the four-digit CIC industry classification, and the year, respectively. Ln(ESI) is the dependent variable, representing the export technological complexity of enterprises. AI is the proxy variable for artificial intelligence, the core explanatory variable in this study. Control_it_ represents a series of control variables, γ_j_ and γ_t_ are fixed effects for industry and year, respectively, and ε_ijt_ is the random error term.

### 3.2. Variable selection

#### 3.2.1. Dependent variable: Export technological complexity of enterprises (ESI).

As high-quality export development has become a focus in recent years, export technological complexity has garnered more attention in academic circles. Following the empirical methods discussed in the chapters, we calculated the export technological complexity of 870 listed companies on the Shanghai and Shenzhen stock exchanges from 2014 to 2021 for comparative analysis. We adopted the approach of Sheng Bin and Mao Qilin (2017) [28] to calculate the export technological complexity as follows. First, calculate the technological complexity of product k:


$$\begin{array}{c} PRODYk=\sum_{c}\frac{(xck/Xc)}{\sum_{c}(xck/Xc)}\times pcgdpc \end{array}$$ (9)

Where PRODY_k_ represents the technological complexity of product k at the HS 6-digit level, x_ck_ represents the export value of product k by country c, X_e_ represents the total export value of all products by country c, and pcgdp_e_ represents the per capita GDP of country c. The first term on the right-hand side represents the revealed comparative advantage of country c in product k.

Adjust the technological complexity of the product for quality:


$$\begin{array}{c} Quilityck=Price\text{\textbackslash{}hspace\{0.33em}\}ck/\sum_{n}(\mu nk\times Price\text{\textbackslash{}hspace\{0.33em}\}nk) \end{array}$$ (10)

$$\begin{array}{c} PROD\overset{adj}{\underset{k}{Y}}=(Quility\text{\textbackslash{}hspace\{0.33em}\}ck{)}^{\lambda }\times PRODYk \end{array}$$ (11)

Where Equation (10) used the unit value of export products to measure product quality. Quility_ck_ represents the export product quality of product k by country c, Price_ck_ represents the export price of the product, and μ_nk_ represents the proportion of product k exported by country n to the total export value of product k worldwide. The higher the ratio, the higher the export product quality. Equation (11) gives the technological complexity of the quality-adjusted product.

Finally, the adjusted product technological complexity is weighted and summed to calculate the export technological complexity of the enterprise:


$$\begin{array}{c} ES\overset{adj}{\underset{i}{I}}=\sum_{k}(\frac{xik}{Xi})\times PROD\overset{adj}{\underset{k}{Y}} \end{array}$$ (12)

Where x_ik_ represents the export value of product k by enterprise i, Xi represents the total export value by enterprise i, and $\overset{\text{adj}}{\underset{\text{i}}{\text{ESI}}}$ is the adjusted product technological complexity. The data is sourced from the UN Comtrade and China Customs Trade databases. The datasets were matched by enterprise name and year, and missing and abnormal values were processed. In the regression model, ESI is logged.

#### 3.2.2. Explanatory variable: Degree of AI adoption (AI).

There is no unified indicator for measuring AI, and different scholars have constructed indicators from various perspectives. Lü Yue (2020) used industrial robot installation density as a proxy for AI to analyze its impact on enterprise global value chain participation [29]. Other studies have used dummy variables for manufacturing companies utilizing AI technologies to examine the impact on productivity.

This study adopts the method of He Qin et al., using the per capita value of machinery and equipment as a proxy for the degree of AI adoption by enterprises, calculated as Ln(book value of machinery/total number of employees), where the book value of machinery is obtained from the fixed asset reports published by enterprises [30]. AI is logged and further standardized in the regression model for subsequent empirical analysis.

#### 3.2.3. Control variables.

Based on existing literature on factors affecting export technological complexity, this study selects twelve indicators from the following four aspects of enterprises as control variables:

1. **Enterprise Development Capability**: Enterprise size (Size), measured as the natural log of the total assets at the end of the period; enterprise market value (Tq), represented by market value/book value.
2. **Enterprise Debt Capacity**: Total asset turnover rate (ATR), represented by the ratio of net sales revenue to average total assets; cash flow ratio (CFR), represented by the ratio of operating cash flow to total liabilities; asset-liability ratio (Lev), represented by the ratio of total liabilities to total assets.
3. **Enterprise Profitability**: Operating expense ratio (OER), represented by the ratio of operating expenses to sales revenue; net profit margin of total assets (ROA), represented by the ratio of total net profit to average total assets; revenue growth rate (RGR), represented by the ratio of revenue growth to revenue in the previous year; capital intensity (FAR), represented by the ratio of fixed assets to total assets.
4. **Enterprise Innovation Capability**: Management expense ratio (MER), represented by the ratio of management expenses to total assets; executive compensation (EI), represented by the natural log of total executive compensation; average age of management (Age), represented by the natural log of the average age of management.

### 3.3. Data sources and descriptive statistics of variables

The data is primarily sourced from the CSMAR database, the WIND database, and the “China Industrial Statistics Yearbook.” Given that the study focuses on industrial robots mainly applied in the manufacturing sector, and based on data availability and timeliness, we set the investigation period from 2014 to 2021, targeting listed manufacturing companies on the Shanghai and Shenzhen stock exchanges for empirical research. After excluding ST and *ST companies and samples with serious data gaps, we obtained a panel dataset of 6960 observations from 870 manufacturing companies. The main continuous variables were subjected to 1% Winsorize processing to eliminate the influence of outliers, and data analysis was conducted using Stata15.0 statistical software. Identify records of enterprise machinery imports from the customs database according to HS codes to obtain data on enterprises that have imported machinery within the sample. Then clean the industrial enterprise database by: (1) removing samples lacking major financial information; and (2) deleting data that do not comply with international general accounting standards. Finally, match the cleaned industrial enterprise database with the machinery import data.Table 1 reports the descriptive statistics of the main variables.

**Table 1:** Descriptive statistics of main variables.

Variable	Note	Obs.	Mean	S.D.	Min	Max
Export Technological Complexity of Enterprises	Ln(ESI)	6960	11.23	0.467	10.00	15.90
AI	Ln(AI)	6960	−0.0230	0.825	−0.839	12.33
Enterprise Scale	Ln(Size)	6960	22.15	1.198	17.64	27.55
Enterprise Market Value	Ln(Tq)	6960	0.571	0.246	0	1.427
Total Asset Turnover Rate	Ln(ATR)	6960	0.615	0.346	0.01	3.513
Cash Flow Ratio	Ln(CFR)	6960	0.0570	0.0640	−0.454	0.488
Asset-Liability Ratio	Ln(Lev)	6960	0.388	0.189	0.0140	0.989
Operating Expense Ratio	Ln(OER)	6960	0.182	0.145	0.00500	1.560
Net Profit Margin of Total Assets	Ln(ROA)	6960	0.0440	0.0700	−0.662	0.478
Revenue Growth Rate	Ln(RGR)	6960	0.198	1.240	−0.864	58.75
Capital Intensity	Ln(FAR)	6960	0.241	0.136	0.00300	0.808
Management Expense Ratio	Ln(MER)	6960	0.0880	0.0660	0.00400	1.436
Executive Compensation	Ln(EI)	6960	15.32	0.966	10.01	18.54
Average Age of Management	Ln(Age)	6960	49.72	3	37.79	61.20

## 4. Empirical analysis

### 4.1. Regression results

Table 2 presents the results of the baseline regression. Stepwise regressions were conducted while controlling for individual and yearly fixed effects. Column (1) shows the regression without including control variables. The coefficient of the core explanatory variable is significantly positive, indicating that Artificial Intelligence (AI) enhances the export technological complexity of manufacturing enterprises at the 1% significance level, thus confirming Hypothesis H1. Columns (2) to (5) display the results of stepwise regressions with the inclusion of control variables from different dimensions. It is evident that as control variables are sequentially added, the coefficient of the core explanatory variable AI gradually decreases, but remains significantly positive at the 1% level, suggesting that the advancement in AI development positively influences the export technological complexity of enterprises. Finally, after including all control variables, the estimated coefficient of the core explanatory variable AI is 0.106. This implies that for each unit increase in AI, the export technological complexity of manufacturing enterprises increases by 0.106 units, further confirming Hypothesis H1. In summary, the enhancement in AI development promotes the increase in export technological complexity, a result verified by the empirical findings of the baseline regression.

**Table 2:** Baseline regression result.

	(1)	(2)	(3)	(4)	(5)
	Ln(ESI)	Ln(ESI)	Ln(ESI)	Ln(ESI)	Ln(ESI)
Ln(AI)	0.234^***^	0.113^***^	0.108^***^	0.110^***^	0.106^***^
	(0.014)	(0.014)	(0.014)	(0.015)	(0.015)
Ln(Size)		0.358^***^	0.342^***^	0.356^***^	0.286^***^
		(0.015)	(0.015)	(0.016)	(0.017)
Ln(Tq)		0.165^***^	0.196^***^	0.151^***^	0.112^***^
		(0.033)	(0.033)	(0.033)	(0.033)
Ln(ATR)			0.155^***^	0.248^***^	0.196^***^
			(0.026)	(0.029)	(0.029)
Ln(CFR)			0.501^***^	0.591^***^	0.493^***^
			(0.104)	(0.105)	(0.104)
Ln(Lev)			0.082	−0.032	0.004
			(0.063)	(0.068)	(0.067)
Ln(OER)				−0.121	0.699^***^
				(0.090)	(0.126)
Ln(ROA)				−0.681^***^	−0.621^***^
				(0.117)	(0.115)
Ln(RGR)				−0.024^***^	−0.017^***^
				(0.005)	(0.005)
Ln(FAR)				−0.196^*^	−0.180^*^
				(0.093)	(0.092)
Ln(MER)					−1.652^***^
					(0.185)
Ln(EI)					0.071^***^
					(0.008)
Ln(Age)					0.022^***^
					(0.004)
_cons	11.231^***^	3.198^***^	3.378^***^	3.189^***^	2.598^***^
	(0.005)	(0.318)	(0.326)	(0.360)	(0.378)
*N*	6960	6960	6960	6960	6960
Industry fixed effects	Yes	Yes	Yes	Yes	Yes
Year fixed effect	Yes	Yes	Yes	Yes	Yes
*R* ^*2*^	0.0409	0.1523	0.1616	0.1705	0.1967

**Note:** ***, **, and * respectively indicate significance at the 1%, 5%, and 10% levels; values in parentheses are t-values; all regressions control for industry fixed effects and year fixed effects.

Regarding the control variables, as the number of control variables increases, the model’s R-squared also rises continuously, indicating the rationality of the control variable selection. Overall, the baseline regression results indicate that the influence of AI development level as the core explanatory variable on export technological complexity of enterprises is positive and significant, and the signs of the control variables are consistent with expectations. This empirical evidence preliminarily supports this study’s theoretical expectations, but the conclusions’ reliability needs to be further validated through robustness tests and other methods.

### 4.2. Mechanism testing

The previous section elaborated on the theoretical pathways through which artificial intelligence affects the export technological complexity of manufacturing enterprises. This section will examine the mechanism by which intelligence affects the export technological complexity of manufacturing enterprises, providing empirical support for theoretical analysis.

Based on the theoretical analysis presented earlier, focusing on the pathways of changing labor structure and enhancing enterprise innovation, the proportion of undergraduate employees in manufacturing enterprise staff is selected as the proxy variable for improving human resource quality from the perspective of labor structure. In contrast, average R&D expenditure per employee is chosen as the proxy variable for enhancing regional innovation from the perspective of increasing R&D investment. Both variables are processed logarithmically. The data are sourced from the Guotaian and Wind databases. The specific descriptions of the proxy variables are provided in Table 3 below.

**Table 3:** Description of mediating variables.

Variable type	Defination	Abbreviation	Measurement Method
Mediating Variable	Labor Structure	talent	Proportion of undergraduate employees in enterprises
	Enterprise Innovation Capability	R&D	Ln(R&D expenditure/ number of employees)

#### 4.2.1. Testing the mechanism of improving labor structure.

The results of testing the mechanism for improving labor structure are shown in Table 4. As indicated in column (1), the regression coefficient of artificial intelligence is positive and significant, confirming that the increase in artificial intelligence does indeed promote the improvement of labor structure. Column (2) demonstrates that, after controlling for the influence of artificial intelligence, the improvement in labor structure significantly enhances the export technological complexity of manufacturing enterprises in China, thereby validating Hypothesis H2 of this study. According to the stepwise regression method, the significance of these two coefficients indicates that labor structure plays a mediating role.

**Table 4:** Testing the mechanism of improving labor structure.

	(1)	(2)
	talent	Ln(ESI)
Ln(AI)	0.710^*^	0.101^***^
	(0.292)	(0.015)
talent		0.004^***^
		(0.001)
Ln(Size)	−0.217	0.297^***^
	(0.344)	(0.017)
Ln(Tq)	3.279^***^	0.102^**^
	(0.661)	(0.033)
Ln(ATR)	0.318	0.199^***^
	(0.587)	(0.030)
Ln(CFR)	9.042^***^	0.451^***^
	(2.092)	(0.106)
Ln(Lev)	−2.295	−0.007
	(1.363)	(0.069)
Ln(OER)	3.701	0.725^***^
	(2.518)	(0.127)
Ln(ROA)	−2.577	−0.589^***^
	(2.301)	(0.116)
Ln(RGR)	−0.131	−0.017^***^
	(0.093)	(0.005)
Ln(FAR)	−2.592	−0.130
	(1.864)	(0.094)
Ln(MER)	−5.079	−1.681^***^
	(3.688)	(0.186)
Ln(EI)	0.179	0.071^***^
	(0.168)	(0.008)
Ln(Age)	−0.017	0.021^***^
	(0.072)	(0.004)
_cons	20.592^**^	2.311^***^
	(7.671)	(0.387)
Industry fixed effects	Yes	Yes
Year fixed effect	Yes	Yes
*N*	6960	6960
*R* ^*2*^	0.0117	0.2016

Note: *, * *, * * * respectively indicate significant levels at 10%, 5%, and 1%; The standard error is in parentheses

#### 4.2.2. Testing the mechanism of enhancing enterprise innovation.

The universality of artificial intelligence, namely its ability to penetrate various fields and realize the concept of “AI+”, has also successfully expanded comprehensive innovation across various sectors. Therefore, following the research methods of previous scholars, this study adopts the logarithm of per capita research and development (R&D) expenditure as a data indicator for the innovation pathway, to examine the role of the innovation pathway in the relationship between artificial intelligence and the export technological complexity of manufacturing enterprises in China. Table 5 indicates that the coefficient of artificial intelligence on innovation is positive and significant, and the impact of innovation on export technological complexity is also significantly positive. This result confirms that artificial intelligence can promote the growth of export technological complexity of manufacturing enterprises in China by improving regional innovation capabilities. As an emerging product of technological revolution, artificial intelligence can drive the transformation and upgrading of more traditional industries and the development of high-tech industries. The flourishing of these industries will inevitably improve the quality and technological content of Chinese products to a certain extent, thereby promoting the growth of export technological complexity of manufacturing enterprises in China.

**Table 5:** Testing the mechanism of enhancing enterprise innovation.

	(1)	(2)
	R&D	Ln(ESI)
Ln(AI)	0.396^***^	0.098^***^
	(0.069)	(0.015)
R&D		0.014^***^
		(0.003)
Ln(Size)	−0.671^***^	0.305^***^
	(0.081)	(0.017)
Ln(Tq)	0.812^***^	0.103^**^
	(0.156)	(0.033)
Ln(ATR)	−0.215	0.203^***^
	(0.138)	(0.030)
Ln(CFR)	0.925	0.472^***^
	(0.492)	(0.106)
Ln(Lev)	0.487	−0.022
	(0.321)	(0.069)
Ln(OER)	2.484^***^	0.705^***^
	(0.593)	(0.127)
Ln(ROA)	−0.102	−0.597^***^
	(0.541)	(0.116)
Ln(RGR)	0.007	−0.018^***^
	(0.022)	(0.005)
Ln(FAR)	−2.092^***^	−0.111
	(0.439)	(0.094)
Ln(MER)	−3.556^***^	−1.652^***^
	(0.868)	(0.186)
Ln(EI)	0.039	0.071^***^
	(0.039)	(0.008)
Ln(Age)	0.005	0.021^***^
	(0.017)	(0.004)
_cons	23.858^***^	2.065^***^
	(1.805)	(0.393)
Industry fixed effects	Yes	Yes
Year fixed effect	Yes	Yes
*N*	6960	6960
*R* ^*2*^	0.0227	0.2005

Note: *, * *, * * * respectively indicate significant levels at 10%, 5%, and 1%; The standard error is in parentheses

### 4.3. Robustness test

The robustness test evaluates the stability of explanatory variables. Three common methods for this test are: changing regression methods, substituting variables, and adjusting data classification. Considering data availability, this study standardized the explanatory variable “level of artificial intelligence adoption” from Ln (machine book value/number of employees) to Ln (machine book value) for representation, as shown in Table 6.

**Table 6:** Robustness test results.

	(1)	(2)
	Ln(ESI)	Ln(ESI)
Ln(AI)		0.106^***^
		(0.015)
Ln(book value of machinery)	0.087^***^	
	(0.012)	
Ln(Size)	0.286^***^	0.286^***^
	(0.017)	(0.017)
Ln(Tq)	0.112^***^	0.112^***^
	(0.033)	(0.033)
Ln(ATR)	0.196^***^	0.196^***^
	(0.029)	(0.029)
Ln(CFR)	0.493^***^	0.493^***^
	(0.104)	(0.104)
Ln(Lev)	0.004	0.004
	(0.067)	(0.067)
Ln(OER)	0.699^***^	0.699^***^
	(0.126)	(0.126)
Ln(ROA)	−0.621^***^	−0.621^***^
	(0.115)	(0.115)
Ln(RGR)	−0.017^***^	−0.017^***^
	(0.005)	(0.005)
Ln(FAR)	−0.181^*^	−0.180^*^
	(0.092)	(0.092)
Ln(MER)	−1.652^***^	−1.652^***^
	(0.185)	(0.185)
Ln(EI)	0.071^***^	0.071^***^
	(0.008)	(0.008)
Ln(Age)	0.022^***^	0.022^***^
	(0.004)	(0.004)
_cons	2.596^***^	2.598^***^
	(0.378)	(0.378)
*N*	6960	6960
Industry fixed effects	Yes	Yes
Year fixed effect	Yes	Yes
R^2^	0.1893	0.1967

Note: *, * *, * * * respectively indicate significant levels at 10%, 5%, and 1%; The standard error is in parentheses.

Table 6 indicates that after replacing the core explanatory variable, the level of artificial intelligence remains significant at the 1% level and has a positive coefficient. This conclusion is consistent with the baseline regression model. Both metrics measuring the level of artificial intelligence show a significant positive effect on the export technological complexity of enterprises, providing evidence for the robustness of this study’s model.

### 4.4. Heterogeneity analysis

#### 4.4.1. Analysis of ownership differentiation.

Given the significant differences between state-owned and non-state-owned enterprises in resource allocation efficiency, enterprise management, and asset scale, especially since state-owned enterprises typically have larger scales, less financing and operational pressure, and relatively lower operational efficiency, we anticipate different responses to the application of artificial intelligence across different ownership enterprises. Therefore, we divided the research sample into two groups, state-owned enterprises, and non-state-owned enterprises, and conducted regression analyses separately. Columns (1) and (2) in Table 7 present the regression results for these two sub-samples. In each regression, multiple control variables, individual fixed effects, and year fixed effects were included to ensure the robustness of the results. From the regression results, we observe that the coefficient of the application of artificial intelligence on the export technological complexity of enterprises is positive for both ownership types, and this effect is more significant for state-owned enterprises. This suggests that the enhancement of artificial intelligence applications has a more pronounced effect on the export technological complexity of state-owned enterprises. The possible reason for this is that the large-scale and relatively lower operational efficiency of state-owned enterprises allows artificial intelligence technology to play a greater role in reducing labor costs and improving efficiency, thereby significantly promoting the increase in the export technological complexity of enterprises.

**Table 7:** Regression results by ownership.

	(1)	(2)
	State-owned enterprises	Non-state-owned enterprises
Ln(AI)	0.113^***^	0.108^***^
	(0.024)	(0.020)
Ln(Size)	0.282^***^	0.273^***^
	(0.036)	(0.021)
Ln(Tq)	0.200^**^	0.052
	(0.061)	(0.039)
Ln(ATR)	0.153^**^	0.187^***^
	(0.055)	(0.035)
Ln(CFR)	0.518^**^	0.400^**^
	(0.191)	(0.125)
Ln(Lev)	−0.214	0.112
	(0.137)	(0.079)
Ln(OER)	0.549	0.661^***^
	(0.327)	(0.142)
Ln(ROA)	−0.243	−0.686^***^
	(0.270)	(0.129)
Ln(RGR)	−0.020^**^	−0.013^*^
	(0.008)	(0.006)
Ln(FAR)	−0.209	−0.065
	(0.172)	(0.114)
Ln(MER)	−1.141^**^	−1.669^***^
	(0.433)	(0.212)
Ln(EI)	0.050^***^	0.119^***^
	(0.009)	(0.017)
Ln(Age)	0.028^***^	0.020^***^
	(0.007)	(0.004)
_cons	2.534^**^	2.317^***^
	(0.818)	(0.447)
*N*	1876	5084
Industry fixed effects	Yes	Yes
Year fixed effect	Yes	Yes
*R* ^*2*^	0.1970	0.2032

Note: *, * *, * * * respectively indicate significant levels at 10%, 5%, and 1%; The standard error is in parentheses.

#### 4.4.2. Analysis of industry differentiation.

Considering that enterprises in different industries have unique technological characteristics, different levels of technological advancement, and differences in research and development investment and innovation capabilities, the ability to absorb technology may affect the export technological complexity of manufacturing enterprises in China. Based on the industry classification in Chapter 3, we conducted an in-depth analysis of heterogeneity in the manufacturing industry and obtained empirical results on the impact of artificial intelligence on the export technological complexity of enterprises in various manufacturing sub-industries, as shown in Table 8. From the regression results, it can be observed that in samples of textile industry, chemical processing industry, and machinery and electronics manufacturing industry, the coefficients of artificial intelligence are 0.196, 0.093, and 0.103 respectively, all significantly positively correlated at the 1% level. This indicates that the advancement of artificial intelligence promotes the export technological complexity of enterprises in the textile industry, chemical processing industry, and machinery and electronics manufacturing industry, with the most significant impact observed in the textile industry. This result may be because different industries have different levels of innovation and varying sensitivity to the application of artificial intelligence. Textile industry enterprises have higher innovation capabilities and stronger learning and absorption capabilities for technology compared to the other two industries. Moreover, textile industry enterprises are more adaptable to increased machine inputs when introducing artificial intelligence technology, thus enabling quicker application in actual production, reducing production costs, improving production efficiency and quality, and consequently increasing the export technological complexity of enterprises and enhancing export competitiveness.

**Table 8:** Regression results by industry.

	(1)	(2)	(3)
	Textile industry	Chemical processing industry	Machinery and electronics manufacturing industry
Ln(AI)	0.196^***^	0.093^***^	0.103^***^
	(0.014)	(0.023)	(0.022)
Ln(Size)	0.233^***^	0.353^***^	0.215^***^
	(0.015)	(0.031)	(0.020)
Ln(Tq)	0.150^***^	0.117	0.070
	(0.024)	(0.063)	(0.037)
Ln(ATR)	0.288^***^	0.215^***^	0.178^***^
	(0.026)	(0.053)	(0.037)
Ln(CFR)	0.421^***^	0.732^***^	0.233^*^
	(0.083)	(0.197)	(0.117)
Ln(Lev)	−0.206^***^	−0.034	0.145
	(0.053)	(0.120)	(0.084)
Ln(OER)	−0.872^***^	0.827^***^	−0.706^*^
	(0.259)	(0.175)	(0.283)
Ln(ROA)	−0.612^***^	−1.011^***^	−0.296^*^
	(0.114)	(0.209)	(0.129)
Ln(RGR)	−0.006^***^	−0.047^**^	−0.026^***^
	(0.002)	(0.015)	(0.008)
Ln(FAR)	−0.546^***^	−0.074	−0.121
	(0.064)	(0.154)	(0.138)
Ln(MER)	0.748^**^	−1.845^***^	−0.629
	(0.280)	(0.316)	(0.354)
Ln(EI)	0.036^***^	0.081^***^	0.051^***^
	(0.007)	(0.013)	(0.013)
Ln(Age)	0.006^*^	0.022^**^	0.022^***^
	(0.003)	(0.007)	(0.004)
_cons	5.050^***^	0.930	4.620^***^
	(0.331)	(0.686)	(0.455)
*N*	1000	3312	2648
Industry fixed effects	Yes	Yes	Yes
Year fixed effect	Yes	Yes	Yes
*R* ^*2*^	0.6893	0.1730	0.2747

Note: *, * *, * * * respectively indicate significant levels at 10%, 5%, and 1%; The standard error is in parentheses

#### 4.4.3. Analysis of regional differentiation.

The regression analysis mentioned above shows that the level of artificial intelligence development significantly promotes the export of technological complexity of enterprises. However, China has unbalanced economic development, and the level of artificial intelligence development varies greatly across different regions. Therefore, it is necessary to consider regional heterogeneity. According to the National Bureau of Statistics division of provinces and referencing the method of dividing economic zones by Li Jianjun et al. (2020), the 870 enterprises were divided into eastern, central, and western regions for sub-sample regression [31].

The sub-sample regression results in Table 9 show that in enterprise samples from the eastern, central, and western regions, the coefficients of artificial intelligence are 0.113, 0.114, and 0.065, respectively, all significantly positively correlated. This indicates that the advancement of artificial intelligence promotes the export technological complexity of enterprises in the eastern, central, and western regions, with the most significant impact observed in the eastern and central regions. This difference may stem from the imbalance in regional development, leading to differences in artificial intelligence development. The eastern coastal region, the most economically developed area in China, is home to many manufacturing and export enterprises, which have a relatively high demand for and application of artificial intelligence technology. Although the central region is rich in resources, its economic development level is slightly lower than that of the eastern region. Therefore, enterprises in the central region need to improve their production efficiency and technological level through the advancement of artificial intelligence. Moreover, the coefficient for the central region is slightly higher than that for the eastern region, indicating that the promotion effect of artificial intelligence application on the export technological complexity of enterprises in the central region is greater. The reason for this is that the application of artificial intelligence reduces labor costs and scale and improves factor allocation efficiency, which is undoubtedly an effective way to enhance the export technological complexity for enterprises in relatively underdeveloped areas. However, enterprises in the eastern region, which already have a high level of export technological complexity and various favorable conditions, may have a relatively limited impact from the enhancement of artificial intelligence. Nevertheless, enterprises in the eastern region can further enhance their independent innovation capabilities by increasing research and development investment and upgrading technology and equipment to expand production and export scale.

**Table 9:** Regression results by region.

	(1)	(2)	(3)
	Eastern region	Central region	West region
Ln(AI)	0.113^***^	0.114^**^	0.065^*^
	(0.020)	(0.041)	(0.031)
Ln(Size)	0.281^***^	0.299^***^	0.288^***^
	(0.020)	(0.047)	(0.051)
Ln(Tq)	0.076^*^	0.182^*^	0.088
	(0.038)	(0.083)	(0.101)
Ln(ATR)	0.163^***^	0.164	0.346^***^
	(0.033)	(0.086)	(0.087)
Ln(CFR)	0.387^**^	0.558^*^	0.766^*^
	(0.119)	(0.275)	(0.315)
Ln(Lev)	−0.041	0.231	−0.114
	(0.079)	(0.182)	(0.190)
Ln(OER)	0.630^***^	0.822^*^	0.968^***^
	(0.162)	(0.340)	(0.282)
Ln(ROA)	−0.594^***^	−0.363	−1.288^***^
	(0.133)	(0.300)	(0.349)
Ln(RGR)	−0.006	−0.039^**^	−0.027^**^
	(0.006)	(0.014)	(0.010)
Ln(FAR)	−0.082	−0.413	−0.028
	(0.112)	(0.268)	(0.232)
Ln(MER)	−1.741^***^	−1.865^***^	−1.332^***^
	(0.237)	(0.520)	(0.392)
Ln(EI)	0.070^***^	0.063^***^	0.279^***^
	(0.011)	(0.015)	(0.047)
Ln(Age)	0.022^***^	0.024^*^	0.013
	(0.004)	(0.010)	(0.011)
_cons	2.796^***^	2.282^*^	−0.363
	(0.443)	(1.047)	(1.200)
*N*	4696	1303	961
Industry fixed effects	Yes	Yes	Yes
Year fixed effect	Yes	Yes	Yes
*R* ^*2*^	0.2043	0.2180	0.2016

Note: *, * *, * * * respectively indicate significant levels at 10%, 5%, and 1%; The standard error is in parentheses.

## 5. Discussion

This paper takes a more refined approach by focusing on the export behavior of micro-level firms. Most existing literature typically relies on macro data provided by the “China Statistical Yearbook,” which only covers analysis at the provincial or industry level within the manufacturing sector, thereby limiting the in-depth exploration of the technological complexity of manufacturing exports from a macro perspective. However, export trade is fundamentally driven by enterprises, and relying solely on aggregated macro data may not accurately capture the impacts on exporting firms in their actual operations, potentially leading to biased research results. Therefore, this paper utilizes the rich data of listed companies from the CSMAR database to explore the impact of artificial intelligence on the technological complexity of exports at the micro-firm level. This shift in research perspective is expected to provide new empirical evidence for a comprehensive understanding of the impact of artificial intelligence on the technological complexity of manufacturing exports.

The impact of artificial intelligence on the economy is an emerging topic that is not yet extensively discussed in academia. While some studies have addressed its effects on economic growth and employment, literature on how artificial intelligence affects the technological complexity of manufacturing firms’ exports remains relatively scarce. This paper aims to establish the connection between artificial intelligence and the technological complexity of manufacturing firms’ exports, not only expanding the application scope of artificial intelligence in the economic field but also enriching the research content on the technological complexity of manufacturing firms’ exports. By thoroughly analyzing the impact and underlying mechanisms of artificial intelligence on the technological complexity of manufacturing firms’ exports, this paper seeks to provide new perspectives and insights for research in related fields.

In our research on this issue, we have strived for comprehensive analysis, but due to certain objective factors, this paper still requires further improvement, such as the specificity of selected indicators. Currently, there is no consensus on the definition of “artificial intelligence,” and it has multifaceted impacts on human economy and society. Based on this, we have chosen alternative variables that better reflect its role in the development of China’s manufacturing firms. However, when analyzing other fields, it is necessary to adopt a multidimensional approach and establish a more comprehensive and diversified measurement system for artificial intelligence, thus more accurately evaluating its role in various aspects.

## 6. Conclusion and policy implications

This study examines the factors influencing the impact of artificial intelligence and the export technological complexity of manufacturing enterprises. The conclusions drawn are as follows:

Firstly, China’s level of artificial intelligence shows a clear three-tier distribution pattern in geographical space. These three tiers belong to the eastern, central, and western provinces, with the level of artificial intelligence decreasing from high to low. Additionally, the level of artificial intelligence in various provinces of China gradually increases over time. There is also significant spatial heterogeneity in export technological complexity, roughly divided into three levels, showing a gradual decrease from east to west and coastal to inland areas. Analysis of the impact of artificial intelligence on enterprise export technological complexity reveals that although both are on the rise, enterprises’ export technological complexity in some provinces fluctuates, indicating that the positive impact of artificial intelligence may not be stable.

Secondly, fixed-effect models will be used to test artificial intelligence’s positive impact on manufacturing enterprises’ export technological complexity. The test results of the baseline regression show that artificial intelligence indeed promotes the export technological complexity of manufacturing enterprises. Furthermore, to ensure the rigor of the empirical analysis, this study also tests alternative indicators for adopting artificial intelligence, and the regression results remain robust.

Thirdly, artificial intelligence has a positive impact on the export technological complexity of manufacturing enterprises, and the theoretical mechanism analysis, this study examines two mechanisms. Enhancing regional innovation has a significant positive effect, indicating that artificial intelligence can improve innovation capabilities and enhance the quality of export products by increasing research and development (R&D) investment. The mechanism of labor structure has a significant positive effect, indicating that artificial intelligence can also increase the technical content of exported goods by changing the labor demand structure, thereby improving manufacturing enterprises’ export technological complexity.

Finally, based on the above research, heterogeneity analysis is conducted by industry, ownership, and region. Because the relationship between industry and the export technological complexity of manufacturing enterprises is closely related, this study further divides the textile, chemical, machinery, and electronics manufacturing industries.Heterogeneity analysis shows that the application of artificial intelligence can significantly promote the improvement of export technological complexity of different categories of enterprises, in which the impact on state-owned enterprises, light textile enterprises and enterprises in the East and Central regions is particularly prominent.

Based on the theoretical analysis and empirical testing of this study, the improvement of artificial intelligence level is an important factor promoting the growth of export technological complexity of manufacturing enterprises. Although the level of artificial intelligence and the export technological complexity of manufacturing enterprises show an upward trend, issues such as low human capital quality, inadequate innovation capabilities, and unbalanced regional development are prominent. Therefore, to maximize the role of artificial intelligence in promoting the growth of export technological complexity of manufacturing enterprises and improving the technological level of China’s manufacturing exports, the following suggestions are proposed:

Firstly, Strengthen research and development and application of artificial intelligence technology. Firstly, the Chinese government should formulate long-term strategies to define artificial intelligence technology’s development direction and application scenarios in the manufacturing industry. Secondly, the government can establish special funds for enterprises and provide incentives such as tax breaks to increase investment. Additionally, manufacturing enterprises should actively explore technological innovation and practical application to promote the deep integration development of production processes.

Secondly, Optimize the labor structure. Firstly, to increase the investment in vocational education and improve its quality and coverage, the government should cooperate with enterprises to jointly formulate training programs to ensure that the training content matches the actual needs while encouraging enterprises to establish internal training systems to provide employees with development opportunities. Secondly, the government should increase the transparency of the labor market information, help workers understand market demand and employment positions comprehensively, establish online recruitment platforms to provide comprehensive employment information and career guidance to workers.

Thirdly, Strengthen enterprise innovation development. Firstly, enterprises should increase investment in innovation. Secondly, enterprises should have a sound innovation system, including establishing specialized research and development institutions, forming innovation teams, etc. Then, enterprises should strengthen cooperation with external innovation resources, such as universities, research institutions, etc., to jointly conduct technological research and development and achieve transformation of results.Finally, enterprises should establish a sound system for protecting and managing intellectual property rights, increase the application and protection of their own intellectual property rights.

Finally, Promote balanced development of artificial intelligence. Local governments need to adapt measures to local conditions and industries according to the actual situation of local development so as to maximize the role of artificial intelligence in promoting the improvement of export technological complexity of manufacturing enterprises.

## Supporting information

S1 File Dataset. (XLSX)
