# Supplementary material for: Research on the impact of artificial intelligence on the export technological complexity of chinese manufacturing enterprises: An analysis based on mediating effects
Source: PLoS One. 2026 Jul 2;21(7):e0351061. doi: 10.1371/journal.pone.0351061 (PMC13327267; doi:10.1371/journal.pone.0351061)
Supplement: Attachment — Submitted filename: Response to Reviwer12.10(1)(2).docx [file pone.0351061.s004.docx]

**Our Response**

Dear Reviewers:

The authors are thankful to the editor and reviewers for their in-depth review and input to improve the manuscript. The manuscript has been substantially refined and improved according to the reviewers' comments. The authors hope this revision has improved the manuscript to the reviewers' and editor's satisfaction level.

Reviewer(s)' Comments to Author:
Reviewer: 1

The comments suggested by reviewer are not related to this paper

Reviewer: 2
Comments:
1. In the abstract section, I have noticed some part that need to be improved to better conform to the writing norms of academic paper abstract.

Thank you for the suggestions. We have revised the abstract according to the expert's feedback and with reference to the journal's format. Please see the updated abstract section for details.

The introduction section of this article lacks logic. This part should mainly include research background, research purpose, literature review, research content and research contribution. But this part does not make the above content clear to the reader.

Thank you for the expert's suggestions. After careful consideration, the authors have completely revised the introduction section of this paper. The specific logical framework for the revisions is as follows:

1. **First Paragraph**: It begins with an overview of the current state and trends of the global industrial revolution, linking it to China's macro policy environment for intelligent transformation and the development and application of industrial robotics. Against this broader context, the research subject of this paper—artificial intelligence (AI)—is introduced. This establishes the significant social and economic value of studying the effects of AI applications.
2. **Second Paragraph**: The theoretical basis is laid out, focusing on how the integration of AI and manufacturing can drive high-quality development in export trade. It identifies export technological complexity as a representative metric for evaluating the high-quality development of manufacturing exports. Additionally, it highlights the growing issue of China's export trade being large in scale but lacking in competitiveness. This serves to introduce the core research question and objective of the paper: how AI can empower the enhancement of export technological complexity and promote the high-quality development of China's manufacturing exports.
3. **Third, Fourth, and Fifth Paragraphs**: These sections provide a literature review. Respectively, they cover the research on the effects of AI applications, the effects of AI applications in the field of international trade, and the effects of AI applications specifically on export technological complexity. Each paragraph acts as a bridge between the preceding and subsequent sections, presenting a logical progression of theoretical developments. Through this structured review, the lack of existing research on the effects of AI on export technological complexity is identified. This reinforces the practical and theoretical significance of the research question and objective, lending the study scientific validity and relevance.
4. **Sixth Paragraph**: This summarizes the marginal contributions (innovative points) of the research, serving as a unifying thread throughout the paper.

This revised introduction ensures a clearer logical flow and emphasizes the significance of the study.

3. Contributions: Although some contributions are listed, but these contributions have been confirmed by a large number of articles, I don't think they belong to the real innovation. I suggest that the authors carefully summarize and refine the innovation points of this study and put forward 2-3 contributions that belong to this paper. In particular, I suggest a comparative analysis with relevant published articles.

Thank you for the suggestions. After careful consideration, the authors have summarized the marginal contributions (innovative points) of this paper into the following three aspects:

1. **Research Perspective and Methodology**:
   While most existing literature evaluates export technological complexity from macro perspectives, such as industry or regional levels, this paper expands the research to the micro-level of individual enterprises. Additionally, it fully considers the potential impact of variations in export product quality when constructing measures of export technological complexity. This effectively addresses the issue of overestimating a country’s or region’s overall export technological complexity level when using HS6-digit product classification. By adopting this research perspective and methodology, the study not only reveals deeper insights into the evolution of export technological complexity within Chinese manufacturing enterprises but also broadens the research scope in the intersection of AI and international trade, offering new pathways and perspectives for subsequent studies in this field.
2. **Exploration of Heterogeneity**:
   This paper goes beyond examining the general effects of AI applications to focus on their differentiated impacts under various micro (ownership structure of enterprises), meso (industry characteristics), and macro (regional disparities) contexts. By providing extensive empirical evidence, this study offers robust support for understanding the specific roles of AI in different types of enterprises, enriching the discourse on the diverse implications of AI applications.
3. **Mechanism Exploration**:
   The study delves into the internal mechanisms through which AI influences enterprise export technological complexity, focusing on two core dimensions: optimizing enterprise labor structures and enhancing their innovation capabilities. This systematic exploration not only enriches the theoretical framework of AI’s effects on export trade but also provides a solid theoretical foundation for future related research.

For further details, please refer to the literature review section.

Literature review: There is no literature review in this paper. I suggest the authors to make a literature summary in the first part of the introduction, analyze the research content of existing literature, and propose the research content of this paper. Finally, this article cites literature that is too old and should cite literature from the last three years. These documents may be helpful for your revision, you can refer to or cite: Green Finance: The Catalyst for Artificial Intelligence and Energy Efficiency in Chinese Urban Sustainable Development. Energy Economics; Is Artificial Intelligence A Curse or A Blessing For Enterprise Energy Intensity? Evidence from China. Energy Economics; Breaking Boundaries: The Influence of Social Insurance Collection System Reform on Corporate Innovation Strategies. Applied Economics.

Thank you for the expert's suggestions. After careful consideration, the authors have conducted a literature review in the first section (Introduction) of the paper. The review is divided into three main aspects:

1. **Research on the Effects of AI Applications**
2. **Research on the Effects of AI Applications in the Field of International Trade**
3. **Research on the Effects of AI Applications Focusing on Export Technological Complexity**

For the cited literature, the primary principle was to select highly relevant works that reflect the historical development of research in each aspect.

5. Data section: This section should clearly tell the reader what data to use and how to handle it.

Thank you for the expert's suggestions. We sincerely apologize for the confusion caused by the lack of clarity regarding the data sources. To address this, we have added details about the data sources used in this paper (see Line#).

Specifically, the data was obtained from the CSMAR database by Guotaian, the WIND database, and the *China Industrial Statistics Yearbook*, which include enterprise-level data. Using Excel, we applied VLOOKUP and other formulas to filter out ST and *ST listed companies, as well as samples with severe data deficiencies. This process resulted in a final dataset comprising a union of 6,960 sample observations from 870 publicly listed manufacturing enterprises.

Additionally, to mitigate the impact of outliers, we Winsorized the main continuous variables at the 1% level at both ends. Data analysis was conducted using Stata 15.0.

6.Results section: The results analysis does not provide an in-depth discussion of its economic implications, that is, the results are not discussed from an economic perspective. including a comparative analysis of the existing literature, specifically in the baseline regression results. This article should add some robustness tests.

Thank you for the expert's suggestions. We have addressed these issues one by one, as detailed below:

1. **Revision of Research Conclusions and Policy Recommendations**:
   In line with the expert's suggestions, we revised the research conclusions and policy recommendations by integrating content from the empirical analysis section. Additionally, we included a discussion section prior to the conclusions to provide further context and insights. For details, please refer to the discussion section in the paper.
2. **Modification of the Explanatory Variable**:
   Based on the expert's feedback and considerations of data availability, we standardized the explanatory variable for AI adoption. Instead of using Ln(machine book value/number of employees), we now represent it as Ln(machine book value). The robustness test results based on this change are presented in the table below.

Table 1: Robustness Test Results

| Variable | (1) | (2) |
| --- | --- | --- |
|  | Ln(ESI) | Ln(ESI) |
| Ln(AI) |  | 0.106*** |
|  |  | (0.015) |
| Ln（book value of machinery） | 0.087*** |  |
|  | (0.012) |  |
| Ln(Size) | 0.286*** | 0.286*** |
|  | (0.017) | (0.017) |
| Ln(Tq) | 0.112*** | 0.112*** |
|  | (0.033) | (0.033) |
| Ln(ATR) | 0.196*** | 0.196*** |
|  | (0.029) | (0.029) |
| Ln(CFR) | 0.493*** | 0.493*** |
|  | (0.104) | (0.104) |
| Ln(Lev) | 0.004 | 0.004 |
|  | (0.067) | (0.067) |
| Ln(OER) | 0.699*** | 0.699*** |
|  | (0.126) | (0.126) |
| Ln(ROA) | -0.621*** | -0.621*** |
|  | (0.115) | (0.115) |
| Ln(RGR) | -0.017*** | -0.017*** |
|  | (0.005) | (0.005) |
| Ln(FAR) | -0.181* | -0.180* |
|  | (0.092) | (0.092) |
| Ln(MER) | -1.652*** | -1.652*** |
|  | (0.185) | (0.185) |
| Ln(EI) | 0.071*** | 0.071*** |
|  | (0.008) | (0.008) |
| Ln(Age) | 0.022*** | 0.022*** |
|  | (0.004) | (0.004) |
| _cons | 2.596*** | 2.598*** |
|  | (0.378) | (0.378) |
| N | 6960 | 6960 |
| Industry fixed effects | Yes | Yes |
| Year fixed effect | Yes | Yes |
| R2 | 0.1893 | 0.1967 |

From Table 1, it can be observed that after replacing the core explanatory variable, the level of AI development remains significant at the 1% level, with a positive coefficient. This result is consistent with the baseline regression model. Both measures of AI development indicate a significant positive impact on the export technological complexity of enterprises, providing strong evidence for the robustness of the model used in this study.

7. Format specification: I recommend the authors to check the full text carefully. At present, the full text has many formatting errors, such as tables, literature citations and so on.

Thank you for the expert's suggestions. We have thoroughly reviewed and revised the language throughout the paper. Additionally, we have made adjustments to the tables, citations, and references. Please refer to the full text for the specific changes.。

Referee: 3

1. The paper is poorly written and there is a lot of Chinglish. The authors should polish the language and make the paper readable.

2. The literature review part in the introduction is messy. Also, the review is not in the correct format. For example,” Gregory et al. believe that using…” should be “Gregory et al. (2016) believe that using…”,” which it does not have an advantage.[13]” should be” which it does not have an advantage [13].”.

Thank you for the expert's suggestion. In this paper, the third, fourth, and fifth paragraphs of the introduction section have been revised to reorganize the literature review. These sections respectively provide:

1. A review of research on the effects of AI applications,
2. A review of research focusing on the effects of AI applications in the field of international trade, and
3. A review of research concentrating on the effects of AI applications on export technological complexity.

Through logical progression and detailed analysis, these reviews lead to the research questions and objectives of this paper. The format of the literature review has been adjusted to align with the review format of *PLOS ONE* (e.g., Hausmann et al., 2007; Goldfarb and Trefler, 2018).

Write all symbols with special meanings (such as j, t, N, P...) in the text using a formula editor.

Thank you for the expert's suggestion. As the expert rightly pointed out, the equations in the methodology section were not formatted using a formula editor. The authors have now re-edited all equations and symbols with special meanings using a formula editor for clarity and accuracy.

3. Please unify the format of Hypothesis H1, Hypothesis H2, and Hypothesis H3.

As the expert rightly pointed out, in the previous version, we noticed an inconsistency in the formatting of Hypothesis H2 compared to the other two hypotheses (H1 and H3). Specifically, H2 was not italicized, unlike the others. We deeply regret any inconvenience this oversight may have caused readers in understanding the text.

To ensure consistency and professionalism in the article, we have made the suggested adjustment and reformatted Hypothesis H2 to italicize it, aligning it with the formatting of the other hypotheses. This revision not only enhances the overall aesthetic quality of the article but also helps readers clearly differentiate and identify each hypothesis.

4. Where is Figure 2-1?

In the original text, we referred to "Figure 2-1" in the hypothesis testing section. However, due to an oversight, the corresponding figure was incorrectly labeled as "Figure 1." This inconsistency may have caused confusion for readers and affected the coherence and readability of the article, for which we sincerely apologize.

To resolve this issue, we have revised the text and corrected the reference to "Figure 2-1," aligning it with the actual label "Figure 1" in the figure. This adjustment ensures the accuracy of the relationship between the text and the figure, helping readers better understand and follow the information presented in the article.

5. What is the "Cathay Pacific listed company" mentioned in the introduction? The article did not mention any relationship with the data analysis in the following text.

Thank you very much for your detailed review of my article and for pointing out the issue regarding the "Cathay Pacific listed company" mentioned in the introduction. Upon careful reflection, we realize that the phrasing in the article may indeed have caused confusion for readers, and we sincerely apologize for this oversight.

The "Cathay Pacific listed company" mentioned in the text actually refers to one of the data sources used for our analysis, specifically the CSMAR database provided by Guotaian. We have clarified this in the revised text to ensure accurate understanding.

6. In the abstract, the data used is from 2008 to 2021, and in page 19, the investigation period is from 2014 to 2021. And why not study until 2022 or 2023?

Thank you very much for your detailed review of my article and for highlighting the inconsistency regarding the data time range as well as the question about the exclusion of 2022 and 2023 data. After a thorough review, we have provided a detailed explanation and clarification.

In the abstract, the data time range is mentioned as 2008 to 2021. This was intended to provide a broader historical context to demonstrate the overall trends of the research topic during this period. However, in the main text on page 19, we specifically describe the investigation and analysis period as 2014 to 2021. This adjustment in the time frame was made based on considerations of data availability and completeness.

Regarding the exclusion of 2022 and 2023 data, we conducted an in-depth discussion. Unfortunately, despite our efforts to collect the most recent data, we found that data from 2022 and 2023 were not sufficiently comprehensive, particularly for the specific indicators and data points critical to our study. Due to the scarcity and incompleteness of this data, we were concerned that including these years might compromise the accuracy and reliability of the results. Therefore, to maintain the rigor and scientific validity of the study, we decided to limit the analysis to data up to 2021.

8. Inconsistent formatting of subheadings in the paper.

Thank you for the expert's suggestion. As the subtitle may cause confusion for both experts and readers, we have removed it.Please refer to the title for details.

9. There is a problem with the reference format.

Thank you for the expert's suggestion. All references in this paper have been standardized and revised to conform to APA format.

10. In the paper, the mediating effect is not reflected in the econometric model. How to measure the innovation capability and labor structure of enterprises?

Thank you very much for your detailed review of my article and for pointing out the concerns regarding the lack of mediation effects in the econometric model, as well as the methods used to measure enterprise innovation capability and labor structure. After careful reflection, we have provided the following detailed explanation and clarification:

**Mediation Effects in the Model**

Your observation regarding the absence of mediation effects is highly insightful. In the initial construction of the article, we focused primarily on analyzing the relationships between the main variables, potentially overlooking the critical role of mediation effects in explaining complex interactions. We acknowledge the significant value of mediation effects in revealing deeper relationships between variables. In future research, we will aim to incorporate mediation effects into the econometric model to provide a more comprehensive exploration and analysis of the relationships between variables.

**Measurement of Enterprise Innovation Capability and Labor Structure**

To measure enterprise innovation capability, we referred to existing literature and selected an indicator that aligns with the specific context and objectives of our study. We used the indicator **"Ln(R&D expenditure/number of employees)"**, which effectively reflects the intensity and efficiency of a company's R&D investment. This is a commonly used and effective measure for assessing enterprise innovation capability.

For labor structure, we adopted the indicator **"proportion of employees with a bachelor's degree or higher"**, which captures the knowledge level and skill structure of the workforce within an enterprise. This measure provides a meaningful reflection of the workforce's educational and skill composition, making it suitable for the purposes of this study.

We appreciate your valuable feedback and will continue to improve our methodology to enhance the robustness and depth of our research.
