# Supplementary material for: Research on the impact of artificial intelligence on the export technological complexity of chinese manufacturing enterprises: An analysis based on mediating effects
Source: PLoS One. 2026 Jul 2;21(7):e0351061. doi: 10.1371/journal.pone.0351061 (PMC13327267; doi:10.1371/journal.pone.0351061)
Supplement: Attachment — Submitted filename: Comments.docx [file pone.0351061.s003.docx]

This paper examines the impact and mechanism of AI on the export technological complexity of Chinese manufacturing enterprises from a corporate perspective. It utilizes data from listed manufacturing companies on the Shanghai and Shenzhen A-shares from 2008 to 2021 and employs a fixed-effects model. After review, it can be seen that the author has made some modifications according to the previous suggestions, but there are still following issues:

1. In 2.2.3 section, the author elaborates on the impact of AI on the export technological complexity of manufacturing enterprises by enhancing innovation capability, and analyzes the influence of AI on the innovation from three aspects. It is suggested to organize these three points into paragraph and use conjunctions such as "firstly", "secondly", and "thirdly".

2. In all regression result tables, the significance of the data is not indicated by an asterisk (*). The capitalization of "yes" and "Yes" in "Table 4-1 Baseline regression result" is not consistent, and there is a lack of relevant explanation for significance identification in the Note.

3. The regression results of the mediation mechanism tests in "Table 4-7" and "Table 4-8" did not control industry and year fixed effects.

4. In“4. Empirical Analysis”section, it is suggested to place "4.3.4 Mechanism Testing" after "4.1 Regression Results".

5. Pay attention to standardizing the article format, indent the first line of all paragraphs, and adjust the font of the "5. Discussion" section.

Overall, the format and language expression of this article need to be further improved.
