# Supplementary material for: Research on the impact of artificial intelligence on the export technological complexity of chinese manufacturing enterprises: An analysis based on mediating effects
Source: PLoS One. 2026 Jul 2;21(7):e0351061. doi: 10.1371/journal.pone.0351061 (PMC13327267; doi:10.1371/journal.pone.0351061)
Supplement: Attachment — Submitted filename: Response to Reviwer2025.02.20.docx [file pone.0351061.s005.docx]

**Our Response**

Dear Reviewers:

The authors are thankful to the editor and reviewers for their in-depth review and input to improve the manuscript. The manuscript has been substantially refined and improved according to the reviewers' comments. The authors hope this revision has improved the manuscript to the reviewers' and editor's satisfaction level.

Reviewer(s)' Comments to Author:
Referee: 2
Comments:

1. In 2.2.3 section, the author elaborates on the impact of AI on the export technological complexity of manufacturing enterprises by enhancing innovation capability, and analyzes the influence of AI on the innovation from three aspects. It is suggested to organize these three points into paragraph and use conjunctions such as "firstly", "secondly", and "thirdly".

[Answer] Thank you for the expert's suggestions. We have revised Section 2.2.3 according to the expert's comments. Please see the updated Section 2.2.3 for details.

2. In all regression result tables, the significance of the data is not indicated by an asterisk (*). The capitalization of "yes" and "Yes" in "Table 4-1 Baseline regression result" is not consistent, and there is a lack of relevant explanation for significance identification in the Note.[Answer] Thank you for the expert's suggestions.

[Answer] Thank you for the expert's suggestions. Due to our oversight, we have caused misunderstanding for the expert and readers, and we sincerely apologize. Following the expert's suggestions, we have added asterisks () to all regression results and included explanatory notes below each regression result table. Additionally, we have standardized the capitalization of "Yes" in all regression results. Please see the revised regression result tables throughout the manuscript for details.

3. The regression results of the mediation mechanism tests in "Table 4-7" and "Table 4-8" did not control industry and year fixed effects.

[Answer] Thank you for the expert's suggestions. Due to the authors' oversight, we have caused confusion for the expert, and we deeply apologize. Based on the expert's suggestions, we have added controls for industry and year fixed effects to the tables.

4.In“4. Empirical Analysis”section, it is suggested to place "4.3.4 Mechanism Testing" after "4.1 Regression Results".

[Answer] Thank you for the expert's suggestions. Following the expert's advice, the authors have moved "4.3.4 Mechanism Testing" to follow "4.1 Regression Results," renumbered it as "4.2," and adjusted the subsequent sections, headings, and table numbers accordingly. Please see the revised fourth section of the article for details.

5. Pay attention to standardizing the article format, indent the first line of all paragraphs, and adjust the font of the "5. Discussion" section.

[Answer] Thank you for the expert's suggestions. The authors have adjusted the formatting throughout the manuscript, particularly ensuring that the first line of all paragraphs is indented. Additionally, the font in the Discussion section has been revised. Please see the full manuscript and the Discussion section for details.

6. Overall, the format and language expression of this article need to be further improved.

[Answer] Thank you for the expert's suggestions. We apologize for the confusion caused by the formatting and language expression issues. Based on the expert's suggestions, we have revised the language and formatting throughout the manuscript.
