# Supplementary material for: Research on the impact of artificial intelligence on the export technological complexity of chinese manufacturing enterprises: An analysis based on mediating effects
Source: PLoS One. 2026 Jul 2;21(7):e0351061. doi: 10.1371/journal.pone.0351061 (PMC13327267; doi:10.1371/journal.pone.0351061)
Supplement: Attachment — Submitted filename: v3_Response to Reviwer(2).docx [file pone.0351061.s006.docx]

**Our Response**

Dear Reviewers:

The authors are thankful to the editor and reviewers for their in-depth review and input to improve the manuscript. The manuscript has been substantially refined and improved according to the reviewers' comments. The authors hope this revision has improved the manuscript to the reviewers' and editor's satisfaction level.

Reviewer(s)' Comments to Author:
Referee: 1
Comments:

1.The introduction of the research question in the paper is a bit sudden; Insufficient literature review on the economic effects of AI (such as neglecting literature on AI and growth like Aghion, and lacking relevant research on China).

Response: Thank you for the suggestion. We have revised the Introduction section to include the research question and a clear research objective at the end (see p. 6). In the second paragraph of the Introduction, foundational theories by Aghion and empirical studies by Chinese scholars have been added to strengthen the theoretical framework on AI’s economic effects (see pp. 4–5). Additionally, the literature gap section has been supplemented to address the "macro-micro linkage gaps" (see p. 5).

2. Figure 1 on page 13 is labeled as Figure 2-1 in the main text, which is incorrect.

Response: Thank you for the expert’s suggestions. We have corrected the figure numbering from Figure 2-1 to Figure 1 as advised (see p. 14).
3. The rationality of AI's proxy variables is insufficient. The paper uses "per capita book value of mechanical equipment (Ln (book value of mechanical equipment/number of employees)" as a proxy variable for AI, but fails to distinguish between traditional machinery and AI technologies such as algorithms and deep learning. It is recommended to use indicators such as the number of enterprise AI patents, the proportion of AI related R&D investment, or industry level robot penetration rate (IFR data) as measurement indicators, which may be more effective.

Response: Thank you for the expert’s suggestions. The indicators recommended—such as the number of enterprise AI patents, the proportion of AI-related R&D investment, and the industry-level robot penetration rate (IFR data)—are theoretically more reasonable and effective for measuring enterprise AI development levels. However, practical challenges exist. Regarding AI patents, there is no comprehensive international disclosure mechanism, and many enterprises do not publicly report such data due to commercial confidentiality or inconsistent data management, making it difficult to obtain high-quality firm-level information. For AI-related R&D investment, inconsistent classification standards and the lack of separate accounting in some firms hinder accurate measurement. The industry-level robot penetration rate (IFR data) reflects only national or industry-wide trends and cannot capture micro-level differences among individual enterprises. Given these limitations and data availability constraints, this study uses the per capita book value of mechanical equipment (Ln [book value of mechanical equipment/number of employees]) as a proxy variable. Although it cannot fully distinguish between traditional machinery and AI technologies, it partially reflects enterprise investment in mechanical equipment, which relates to technological upgrades and AI applications. Future research will explore more suitable indicators as data conditions improve.
4. Additional explanation is needed for the calculation of export technology complexity (ESI). The weighting method for enterprise level ESI in formula (12) is reasonable, but it does not explain how to handle the matching problem between product classification (HS 6-digit code) and enterprise products. Suggestion: Supplement the details of the data matching process (such as the docking method between customs data and enterprise export products) to avoid selective bias.

Response: Thank you for the expert’s suggestions. A detailed explanation of the data matching process has been added to the last paragraph of Section 3.2.1 (see p. 16).

5.The policy recommendations lack specificity, and the correlation between policy recommendations and empirical findings should be strengthened.

Response: Thank you for the expert’s suggestions. The policy recommendations section has been revised accordingly (see pp. 32–34).

6. The reference format is inconsistent. Some literature lacks page numbers (such as [24]), and some are cited repeatedly (such as [13] and [14] both by Dixit & Stiglitz, 1977).

Response: Thank you for the expert’s suggestions. Upon review, we identified errors in the literature citations. The original reference [24] has been updated to [30] after reordering. Duplicate citations [13] and [14] have been removed, and all references have been renumbered accordingly throughout the paper.

Referee: 4
Comments:

1. In the introduction, please clearly articulate the contributions of this research.

Response: Thank you for the expert’s suggestions. The contributions of this research have been clearly articulated in the Introduction section (see p. 6).

2. Key literature should be incorporated into the literature review, such as:
DOI: 10.1109/TEM.2025.3543210
doi.org/10.1016/j.jebo.2023.05.008
doi.org/10.1016/j.jfineco.2023.103745

Response: Thank you for the expert’s suggestions. Key literature has been added to the literature review section (see pp. 5–6), and the references have been updated accordingly.

3. Why are firm fixed effects not included in the main regression model? Controlling for firm fixed effects could enhance the model's explanatory power.

Response: Thank you for the expert’s suggestions. The main regression model does not include firm fixed effects for the following reasons:

Sample enterprises are grouped into three major industry categories based on their characteristics, and industry fixed effects are included in the model.

Theoretically, while firm fixed effects control for time-invariant firm-level traits (e.g., corporate culture, management style), these are not the primary determinants of the relationship studied here. Industry-level factors such as technological development, market competition, and policy environment more directly influence key outcomes like export technology complexity. Controlling for industry fixed effects better captures industry heterogeneity and common shocks, allowing clearer identification of the core explanatory variables’ effects.

Empirically, including firm fixed effects would greatly reduce degrees of freedom given the large sample size and time span, risking multicollinearity and less stable estimates. Industry fixed effects offer a better balance between degrees of freedom, precision, and explanatory power.

Future research with larger samples and improved data structures may explore adding both firm and industry fixed effects to enhance rigor and control for more factors comprehensively.

4. Please carefully proofread and check for grammatical issues; further language polishing is necessary. References 12 and 13 are duplicates.

Response: Thank you for the experts’ suggestions. We have proofread the paper and duplicate reference has been removed.

7. PLOS authors have the option to publish the peer review history of their article ([what does this mean?](https://track.editorialmanager.com/CL0/https://journals.plos.org/plosone/s/editorial-and-peer-review-process%23loc-peer-review-history/1/010f019757c2c851-48814a98-5a9e-479f-b55b-a36018ee1001-000000/OTtJ4N30bngRQ8w-kqAD09N1Wzsn7AYOxCXrM8K5UiE=214)). If published, this will include your full peer review and any attached files.
Response: This means that when authors publish their paper with PLOS journals, they can choose to make the entire peer review process publicly available. This includes all the reviewer comments, author responses, editorial decisions, and any files exchanged during review. It adds transparency by letting readers see the discussion and revisions behind the final published article. We agree with the expert's recommendation.
