# Supplementary material for: Research on the impact of artificial intelligence on the export technological complexity of chinese manufacturing enterprises: An analysis based on mediating effects
Source: PLoS One. 2026 Jul 2;21(7):e0351061. doi: 10.1371/journal.pone.0351061 (PMC13327267; doi:10.1371/journal.pone.0351061)
Supplement: Attachment — Submitted filename: Response.docx [file pone.0351061.s007.docx]

**Research on the Impact of Artificial Intelligence on the Export Technological Complexity of Chinese Manufacturing Enterprises: An Analysis Based on Mediating Effects**

Revision Notes

Dear Esteemed Reviewer：

Thank you very much for taking time out of your busy schedule to review our manuscript and for providing valuable revision suggestions, which have significantly contributed to improving the quality of our work. The authors have carefully examined the reviewer's comments, consulted additional literature, furthered our understanding, broken down the suggestions in detail, and revised the paper meticulously word by word.

Below, we address each of the reviewer's comments point by point:

Reviewer 1 Comments:

Comment 1:The literature review section lacks key references and suffers from insufficient logical clarity. Specific issues include: (1) The review of research on the economic effects of artificial intelligence is not systematic enough, omitting fundamental theoretical works, such as studies by Aghion et al. on AI and economic growth, as well as important empirical research on AI and firm behavior in the Chinese context; (2) The review fails to clearly delineate the gaps in existing research and correspondingly highlight the marginal contribution of this study.

**Revision Explanation:**

The following two modifications have been made to the literature review section:

（1）Key references have been added. Theoretical foundations now include studies by Aghion et al. (2017), Koch et al. (2021), and Brynjolfsson et al. (2019) on AI and economic growth and the labor market.
（2）The research gap and marginal contribution of the paper have been clearly articulated.

Summary of Revisions:

Please refer to the sections highlighted in red in the manuscript for your review.

Comment 2:The theoretical mechanism analysis is insufficiently deep. Although two mediating mechanisms, "labor structure optimization" and "innovation capability enhancement," are proposed, the theoretical analysis section (Chapter 2) lacks in-depth elaboration on the micro-mechanisms through which AI affects export technical sophistication via these two pathways. It also lacks rigorous theoretical derivation and literature support.

**Revision Explanation:**

The theoretical mechanisms section in Chapter 2 has been systematically deepened, with key additions of relevant literature including Yang Xiaoxia et al. (2024), Chen Jia et al. (2023), Zhao Chunming et al. (2025), and Jin Zehu et al. (2023). These provide a more solid theoretical basis and empirical support for the two transmission pathways of labor structure optimization and innovation capability enhancement. The revised mechanism analysis features more rigorous logic and more sufficientargumentation

Summary of Revisions:

Please refer to the sections highlighted in red in the manuscript for your review.

Comment 3:The measurement of the core explanatory variable (Artificial Intelligence AI) is not sufficiently reasonable. The paper uses "per capita book value of machinery and equipment" as a proxy variable for AI, which has significant defects. It fails to distinguish between traditional machinery and AI technologies involving algorithms and deep learning, thus making it difficult to accurately reflect the firm's "artificial intelligence" level. This is prone to measurement error and endogeneity issues. Alternatives include using industry-level robot penetration rates (IFR data) matched to the firm's industry, or using the number of AI-related patent applications/grants by firms. Additionally, text analysis methods can be employed to extract the frequency of AI keywords from annual reports as a measurement approach.

**Revision Explanation:**

Existing literature has used industry-level robot penetration rates (IFR data) matched to the industry of firms to measure AI levels.

Summary of Revisions:

Please refer to the sections highlighted in red in the manuscript for your review.

Comment 4:The main regression model only controls for industry and year fixed effects, without controlling for firm fixed effects. Consequently, it cannot rule out the influence of firm heterogeneity (e.g., corporate culture, management capabilities) on the estimation results.

**Revision Explanation:**

Heterogeneity analysis was conducted by dividing firms into state-owned and non-state-owned enterprises.

Summary of Revisions:

Please refer to the sections highlighted in red in the manuscript for your review.

Comment 5:The policy recommendations are too general and lack strong relevance to the empirical results. The suggestions in the policy recommendation section are relatively vague (e.g., "strengthen AI technology R&D," "optimize labor structure") and are not closely integrated with the empirical findings of this paper (e.g., heterogeneity results: more significant effects in state-owned enterprises and the eastern and central regions; mediating mechanisms: the roles of labor structure and innovation) to propose precise and actionable policy solutions.

**Revision Explanation:**

(I) Precise Measures Based on Benchmark Effects and Mediating Mechanisms

Policy Recommendations Targeting the "Labor Structure Optimization" Mediating Pathway:

Firm Level: Implement "AI Skills Upgrade Programs," collaborate with vocational schools and AI technology companies to provide targeted training on intelligent equipment operation, data processing, etc., for existing low-skilled employees; establish "High-Skilled Talent Incentive Mechanisms" to improve compensation and career advancement pathways for AI-related positions, attracting external high-skilled talent.

Government Level: Provide financial subsidies for AI skills training conducted by firms; build "AI Talent Training Bases" in manufacturing-intensive regions, focusing on cultivating practical skilled talent that meets enterprise needs; improve the vocational education system by adding majors such as Artificial Intelligence and Industrial Automation to alleviate the shortage of high-skilled talent.

Policy Recommendations Targeting the "Innovation Capability Enhancement" Mediating Pathway:

Firm Level: Increase AI R&D investment, focusing on AI technology R&D related to core products (e.g., intelligent production algorithms, AI systems for product quality inspection); strengthen collaboration with universities and research institutes to establish joint AI innovation laboratories, accelerating the commercialization of technological achievements.

Government Level: Establish a "Manufacturing AI Innovation Special Fund" to provide financial support for firms' AI R&D projects; optimize the intellectual property protection system, strengthening the protection of AI-related patents; build an "AI Technology Sharing Platform" to facilitate the transfer and diffusion of AI technological achievements from universities and research institutes to SMEs.

(II) Differentiated Policy Recommendations Based on Heterogeneity Results

Policy Recommendations Targeting State-Owned Enterprises (SOEs):

Leverage the exemplary and leading role of SOEs by supporting them in building "AI + Manufacturing" benchmark projects (e.g., smart factories, digital twin production lines) to provide replicable experiences for the industry; encourage SOEs to engage in mixed-ownership cooperation with private AI enterprises to avoid "reinventing the wheel" and enhance the efficiency of market-oriented application of AI technology.

Policy Recommendations Targeting Regional Disparities:

Eastern Region: Focus on building AI industry clusters, supporting cross-enterprise and cross-regional collaborative innovation in AI technology; promote the deep integration of AI with high-end manufacturing, focusing on high-tech export sectors such as semiconductors and precision machinery to further enhance export technical sophistication.

Central Region: Leverage the advantage of undertaking industrial relocation, focus on promoting mature AI application technologies (e.g., industrial robots, intelligent logistics systems) to accelerate the intelligent transformation of traditional manufacturing; strengthen technological cooperation with the eastern region to introduce advanced AI technologies and talents.

Western Region: Prioritize improving digital infrastructure (e.g., 5G networks, Industrial Internet) to lower the hardware threshold for AI application; conduct "AI Technology Popularization Training" to help enterprise managers and technicians enhance their understanding and application capabilities of AI; grant enterprises in the western region more favorable AI R&D subsidies and tax reductions to narrow regional disparities.

Summary of Revisions:

Please refer to the sections highlighted in red in the manuscript for your review.

Reviewer 2 Comments:

Comment 1:In 2.2.3 section, the author elaborates on the impact of AI on the export technological complexity of manufacturing enterprises by enhancing innovation capability, and analyzes the influence of AI on the innovation from three aspects. It is suggested to organize these three points into paragraph and use conjunctions such as "firstly", "secondly", and "thirdly".

**Revision Explanation:**

Thank you for your suggestion. In the revised manuscript, the three aspects regarding AI's influence on innovation capability have been reorganized into a coherent paragraph using the conjunctions "firstly," "secondly," and "thirdly" as recommended.

Summary of Revisions:

Please refer to the sections highlighted in red in the manuscript for your review.

Comment 2:In all regression result tables, the significance of the data is not indicated by an asterisk (*). The capitalization of "yes" and "Yes" in "Table 4-1 Baseline regression result" is not consistent, and there is a lack of relevant explanation for significance identification in the Note.

**Revision Explanation:**

Your point regarding the table notations is crucial. All regression tables have been updated to include asterisks indicating statistical significance, the notation for "Yes" has been standardized, and a clear explanation of significance levels has been added to the notes.

Summary of Revisions:

Please refer to the sections highlighted in red in the manuscript for your review.

Comment 3:The regression results of the mediation mechanism tests in "Table 4-7" and "Table 4-8" did not control industry and year fixed effects.

**Revision Explanation:**

Regarding the control variables in the mechanism test tables, we have carefully reviewed and re-ran the regression analyses. The models in Tables 4-7 and 4-8 now include controls for both industry and year fixed effects.

Summary of Revisions:

Please refer to the sections highlighted in red in the manuscript for your review.

Comment 4:In“4. Empirical Analysis”section, it is suggested to place "4.3.4 Mechanism Testing" after "4.1 Regression Results".

**Revision Explanation:**

Following your feedback on the paper's structure, we have moved the "4.3.4 Mechanism Testing" section to follow "4.1 Regression Results" to improve the logical flow of the empirical analysis.

Summary of Revisions:

Please refer to the sections highlighted in red in the manuscript for your review.

Comment 5:Pay attention to standardizing the article format, indent the first line of all paragraphs, and adjust the font of the "5. Discussion" section.

**Revision Explanation:**

Thank you for your attention to the formatting details. The first line of all paragraphs has been properly indented, and the font in the "5. Discussion" section has been adjusted to conform to the required style guidelines.

Summary of Revisions:

Please refer to the sections highlighted in red in the manuscript for your review.

We tried our best to improve the manuscript and made some changes. These changes will not influence the content and framework of the paper. Once again, thank you very much for your comments and suggestions.

Thank you once again to the esteemed reviewers for your valuable comments!

We wish you all the very best of health and every success!

Yours sincerely,
Mengtian Wang
