# Supplementary material for: Research on the impact of artificial intelligence on the export technological complexity of chinese manufacturing enterprises: An analysis based on mediating effects
Source: PLoS One. 2026 Jul 2;21(7):e0351061. doi: 10.1371/journal.pone.0351061 (PMC13327267; doi:10.1371/journal.pone.0351061)
Supplement: Peer review history

# Peer review: Decision Letter 0

**DOI:** 10.1371/journal.pone.0351061.r001

**Authors:** Van Thanh Tien Nguyen

29 Oct 2024

PONE-D-24-35896

Research on the Impact of Artificial Intelligence on the Export Technological Complexity of Chinese Manufacturing Enterprises: An Analysis Based on Mediating Effects

PLOS ONE

Dear Dr. Li,

Thank you for submitting your manuscript to PLOS ONE. After careful consideration, we feel that it has merit but does not fully meet PLOS ONE’s publication criteria as it currently stands. Therefore, we invite you to submit a revised version of the manuscript that addresses the points raised during the review process.

Please submit your revised manuscript by Dec 13 2024 11:59PM. If you will need more time than this to complete your revisions, please reply to this message or contact the journal office at plosone@plos.org. When you're ready to submit your revision, log on to https://www.editorialmanager.com/pone/ and select the 'Submissions Needing Revision' folder to locate your manuscript file.

Please include the following items when submitting your revised manuscript:

- A rebuttal letter that responds to each point raised by the academic editor and reviewer(s). You should upload this letter as a separate file labeled 'Response to Reviewers'.
- A marked-up copy of your manuscript that highlights changes made to the original version. You should upload this as a separate file labeled 'Revised Manuscript with Track Changes'.
- An unmarked version of your revised paper without tracked changes. You should upload this as a separate file labeled 'Manuscript'.

If you would like to make changes to your financial disclosure, please include your updated statement in your cover letter. Guidelines for resubmitting your figure files are available below the reviewer comments at the end of this letter.

If applicable, we recommend that you deposit your laboratory protocols in protocols.io to enhance the reproducibility of your results. Protocols.io assigns your protocol its own identifier (DOI) so that it can be cited independently in the future. For instructions see: https://journals.plos.org/plosone/s/submission-guidelines#loc-laboratory-protocols. Additionally, PLOS ONE offers an option for publishing peer-reviewed Lab Protocol articles, which describe protocols hosted on protocols.io. Read more information on sharing protocols at https://plos.org/protocols?utm_medium=editorial-email&utm_source=authorletters&utm_campaign=protocols.

We look forward to receiving your revised manuscript.

Kind regards,

Van Thanh Tien Nguyen, Ph.D.

Academic Editor

PLOS ONE

Journal Requirements:

1. When submitting your revision, we need you to address these additional requirements.

Please ensure that your manuscript meets PLOS ONE's style requirements, including those for file naming. The PLOS ONE style templates can be found at

https://journals.plos.org/plosone/s/file?id=wjVg/PLOSOne_formatting_sample_main_body.pdf and

https://journals.plos.org/plosone/s/file?id=ba62/PLOSOne_formatting_sample_title_authors_affiliations.pdf

2. Thank you for stating the following financial disclosure: “This work was supported by National Social Science Fund of China [grant number 23BGL204]; Program for Young Talents of Science and Technology in Universities of the Inner Mongolia Autonomous Region [grant numbers NJYT22113]; Inner Mongolia University Direct Scientific Research Business Fee Project [grant numbers NCYWR22020, NCYWR22021];"Five Major Tasks" Special Research Project of Inner Mongolia University of Finance and Economics in 2024(NCXWD2403)”.

Please state what role the funders took in the study.  If the funders had no role, please state: "The funders had no role in study design, data collection and analysis, decision to publish, or preparation of the manuscript."

If this statement is not correct you must amend it as needed.

Please include this amended Role of Funder statement in your cover letter; we will change the online submission form on your behalf.

3. Thank you for stating the following in the Acknowledgments Section of your manuscript: “This work was supported by National Social Science Fund of China [grant number 23BGL204]; Program for Young Talents of Science and Technology in Universities of the Inner Mongolia Autonomous Region [grant numbers NJYT22113]; Inner Mongolia University Direct Scientific Research Business Fee Project [grant numbers NCYWR22020, NCYWR22021]；"Five Major Tasks" Special Research Project of  Inner Mongolia University of Finance and Economics in 2024(NCXWD2403).”

We note that you have provided funding information that is not currently declared in your Funding Statement. However, funding information should not appear in the Acknowledgments section or other areas of your manuscript. We will only publish funding information present in the Funding Statement section of the online submission form.

Please remove any funding-related text from the manuscript and let us know how you would like to update your Funding Statement. Currently, your Funding Statement reads as follows: “This work was supported by National Social Science Fund of China [grant number 23BGL204]; Program for Young Talents of Science and Technology in Universities of the Inner Mongolia Autonomous Region [grant numbers NJYT22113]; Inner Mongolia University Direct Scientific Research Business Fee Project [grant numbers NCYWR22020, NCYWR22021];"Five Major Tasks" Special Research Project of Inner Mongolia University of Finance and Economics in 2024(NCXWD2403).”

Please include your amended statements within your cover letter; we will change the online submission form on your behalf.

4. PLOS requires an ORCID iD for the corresponding author in Editorial Manager on papers submitted after December 6th, 2016. Please ensure that you have an ORCID iD and that it is validated in Editorial Manager. To do this, go to ‘Update my Information’ (in the upper left-hand corner of the main menu), and click on the Fetch/Validate link next to the ORCID field. This will take you to the ORCID site and allow you to create a new iD or authenticate a pre-existing iD in Editorial Manager.

Additional Editor Comments:

Dear Dr. Li,

Thank you for contributing to Plos One.

One or more of the reviewers has recommended that you cite specific previously published works. Members of the editorial team have determined that the works referenced are not directly related to the submitted manuscript. As such, please note that it is not necessary or expected to cite the works requested by the reviewer.

Thank you for your attention.

Best regards,

Van Thanh Tien Nguyen, Ph.D.

Academic Editor,

Plos ONE.

[Note: HTML markup is below. Please do not edit.]

Reviewers' comments:

Reviewer's Responses to Questions

**Comments to the Author**

1. Is the manuscript technically sound, and do the data support the conclusions?

The manuscript must describe a technically sound piece of scientific research with data that supports the conclusions. Experiments must have been conducted rigorously, with appropriate controls, replication, and sample sizes. The conclusions must be drawn appropriately based on the data presented.

Reviewer #1: Yes

Reviewer #2: Yes

Reviewer #3: Partly

**********

2. Has the statistical analysis been performed appropriately and rigorously?

Reviewer #1: Yes

Reviewer #2: Yes

Reviewer #3: I Don't Know

**********

3. Have the authors made all data underlying the findings in their manuscript fully available?

The PLOS Data policy requires authors to make all data underlying the findings described in their manuscript fully available without restriction, with rare exception (please refer to the Data Availability Statement in the manuscript PDF file). The data should be provided as part of the manuscript or its supporting information, or deposited to a public repository. For example, in addition to summary statistics, the data points behind means, medians and variance measures should be available. If there are restrictions on publicly sharing data—e.g. participant privacy or use of data from a third party—those must be specified.

Reviewer #1: Yes

Reviewer #2: Yes

Reviewer #3: Yes

**********

4. Is the manuscript presented in an intelligible fashion and written in standard English?

PLOS ONE does not copyedit accepted manuscripts, so the language in submitted articles must be clear, correct, and unambiguous. Any typographical or grammatical errors should be corrected at revision, so please note any specific errors here.

Reviewer #1: Yes

Reviewer #2: Yes

Reviewer #3: Yes

**********

5. Review Comments to the Author

Please use the space provided to explain your answers to the questions above. You may also include additional comments for the author, including concerns about dual publication, research ethics, or publication ethics. (Please upload your review as an attachment if it exceeds 20,000 characters)

Reviewer #1: This paper studies the impact of corporate carbon disclosure level on the carbon disclosure behavior of individual enterprises in the same industry and the moderating effect of media attention, which is innovative. This paper finds that there is a peer effect in carbon information disclosure in the same industry. The level of carbon disclosure of enterprises in the same industry has a significant positive impact on the level of carbon disclosure of individual enterprises, and media attention plays a significant positive regulatory role in this relationship. In addition, this paper also finds that there is a significant difference in peer influence between environmentally sensitive industries and non-environmentally sensitive industries, which enriches the relevant research on carbon information disclosure, and is conducive to providing reference for enterprises to adopt environmental strategies, strengthen industry supervision and improve relevant policies for the government. Before considering publication, the paper has some problems that must be solved :

1.In the Abstract section, it is recommended to modify the expression :

(1) The existing expression 'Due to the escalating severity of global climate change. Nations throughout the world are actively supporting a low-carbon economy. Carbon Information Disclosure (CID) has become a venue for companies to demonstrate their efforts to decrease carbon emissions to the public.' It cannot clearly tell readers why to study this topic.

(2) 'The pertinent findings furnish policymakers and business with more thorough and useful understandings. Establish a robust empirical foundation for enhancing industry regulation and media collaboration, and aid in the development of more pragmatic policies and strategies.' The main research significance of this study has not been written, and the expression is too vague.

2. In the Introduction section :

(1) In the policy background, the first paragraph is more a simple list of China 's policies in different periods, but in fact it does not explain the policy meaning clearly; at the same time, the first paragraph only shows that 'Enterprises, being the primary source of carbon emissions, are under pressure to reduce emissions from the outside world in this situation.' and 'carbon disclosure is regarded as a significant means for corporations to engage with external stakeholders and long-term relationships'. Moreover, it is emphasized that 'However, a standardized framework for carbon information disclosure has not been established yet.', and then concluded that the research topic of this paper is very valuable. However, the argument of this conclusion is insufficient.

(2) In the theoretical background, starting from the second paragraph, the peer effect is first explained by nouns, and then the reasons for the peer effect of enterprises are explained based on the signal theory. Secondly, the role of peer effect in enterprise decision-making and the role of information disclosure in enterprise decision-making are mentioned. Finally, the carbon disclosure is contacted to explain the reasons for the peer effect. The upper and lower connections between these theoretical analyses are not enough, and they are relatively scattered. It is suggested to think about how to sort out the logic of these paragraphs to make them clearer. At the same time, at present, these paragraphs have not enough connection with the theme of carbon information disclosure, and it is suggested to strengthen the connection with the theme in writing.

(3) The sixth paragraph describes the peer effect of carbon information disclosure. However, the logic is not clear and the content is less about how carbon information disclosure affects enterprises. It is suggested to sort out the impact mechanism.

(4) Among the research contributions, the third research contribution is about the lag test. However, in the following 4.5.2 Lag test, only a short paragraph is used to describe the contribution, and there is no description of the use of this method in other articles in the past, and the argument is seriously insufficient.

3. In the Theoretical analysis and research hypotheses part:

(1) It is suggested that the proper nouns of the whole text should be consistent. For example, 'dynamic competition theory' and 'competition theory' should use the same proper nouns.

(2) In the literature review and hypothesis analysis related to hypothesis 2, it focuses on how media attention affects corporate information disclosure, but as a moderating variable, how media attention affects the relationship between the level of carbon disclosure of enterprises in the same industry and the level of carbon disclosure of individual enterprises. The description of the impact mechanism is not enough. Please supplement this and sort out the logical relationship.

4. In the 4.4 Heterogeneity analysis section, the author conducted a heterogeneity analysis of environmentally sensitive industries and environmentally insensitive industries, and concluded that 'although the impact of peer influence on carbon information disclosure is considerable for both types of companies. Obviously it is clear that the influence is more pronounced in industries that are not environmentally sensitive.', but the reasons and internal mechanism are too rough to be added by the author.

5. There are many problems in the format of the article. The author is asked to carefully correct the format problems according to the relevant standards, including the reference part.

In a word, the paper carries out a main regression analysis, a moderating effect analysis and a heterogeneity analysis. On the whole, the content is simple and not rich enough, and lacks in-depth analysis and discussion. At the same time, the logic of some paragraphs is not strong enough, and it is not closely combined with the research topic of carbon information disclosure in this paper.

Reviewer #2: This paper examines the impact and mechanism of AI on the export technological complexity of Chinese manufacturing enterprises from a corporate perspective. This paper is interesting but not well written. I recommend a major revision for further consideration.

1. In the abstract section, I have noticed some part that need to be improved to better conform to the writing norms of academic paper abstract.

2. The introduction section of this article lacks logic. This part should mainly include research background, research purpose, literature review, research content and research contribution. But this part does not make the above content clear to the reader.

3. Contributions: Although some contributions are listed, but these contributions have been confirmed by a large number of articles, I don't think they belong to the real innovation. I suggest that the authors carefully summarize and refine the innovation points of this study and put forward 2-3 contributions that belong to this paper. In particular, I suggest a comparative analysis with relevant published articles.

4. Literature review: There is no literature review in this paper. I suggest the authors to make a literature summary in the first part of the introduction, analyze the research content of existing literature, and propose the research content of this paper. Finally, this article cites literature that is too old and should cite literature from the last three years. These documents may be helpful for your revision, you can refer to or cite: Green Finance: The Catalyst for Artificial Intelligence and Energy Efficiency in Chinese Urban Sustainable Development. Energy Economics; Is Artificial Intelligence A Curse or A Blessing For Enterprise Energy Intensity? Evidence from China. Energy Economics; Breaking Boundaries: The Influence of Social Insurance Collection System Reform on Corporate Innovation Strategies. Applied Economics.

5. Data section: This section should clearly tell the reader what data to use and how to handle it.

6. Results section: The results analysis does not provide an in-depth discussion of its economic implications, that is, the results are not discussed from an economic perspective. including a comparative analysis of the existing literature, specifically in the baseline regression results. This article should add some robustness tests.

7. Format specification: I recommend the authors to check the full text carefully. At present, the full text has many formatting errors, such as tables, literature citations and so on.

8.

Reviewer #3: In the manuscript, the authors examined the impact and mechanism of AI on the export technological complexity of Chinese manufacturing enterprises from a corporate perspective. I have the following comments that hope the authors could consider. Major revision is required

1. The paper is poorly written and there is a lot of Chinglish. The authors should polish the language and make the paper readable.

2. The literature review part in the introduction is messy. Also, the review is not in the correct format. For example,” Gregory et al. believe that using…” should be “Gregory et al. (2016) believe that using…”,” which it does not have an advantage.[13]” should be” which it does not have an advantage [13].”.

3. Write all symbols with special meanings (such as j, t, N, P...) in the text using a formula editor.

4. Please unify the format of Hypothesis H1, Hypothesis H2, and Hypothesis H3.

5. Where is Figure 2-1?

6. What is the "Cathay Pacific listed company" mentioned in the introduction? The article did not mention any relationship with the data analysis in the following text.

7. In the abstract, the data used is from 2008 to 2021, and in page 19, the investigation period is from 2014 to 2021. And why not study until 2022 or 2023?

8. Inconsistent formatting of subheadings in the paper.

9. There is a problem with the reference format.

10. In the paper, the mediating effect is not reflected in the econometric model. How to measure the innovation capability and labor structure of enterprises?

**********

6. PLOS authors have the option to publish the peer review history of their article (what does this mean?). If published, this will include your full peer review and any attached files.

If you choose “no”, your identity will remain anonymous but your review may still be made public.

**Do you want your identity to be public for this peer review?**  For information about this choice, including consent withdrawal, please see our Privacy Policy.

Reviewer #1: **Yes:** Yongjun Tang

Reviewer #2: No

Reviewer #3: No

**********

[NOTE: If reviewer comments were submitted as an attachment file, they will be attached to this email and accessible via the submission site. Please log into your account, locate the manuscript record, and check for the action link "View Attachments". If this link does not appear, there are no attachment files.]

While revising your submission, please upload your figure files to the Preflight Analysis and Conversion Engine (PACE) digital diagnostic tool, https://pacev2.apexcovantage.com/. PACE helps ensure that figures meet PLOS requirements. To use PACE, you must first register as a user. Registration is free. Then, login and navigate to the UPLOAD tab, where you will find detailed instructions on how to use the tool. If you encounter any issues or have any questions when using PACE, please email PLOS at figures@plos.org. Please note that Supporting Information files do not need this step.

# Author comment: Author response to Decision Letter 1

**DOI:** 10.1371/journal.pone.0351061.r002

26 Dec 2024

Our Response

Dear Reviewers:

The authors are thankful to the editor and reviewers for their in-depth review and input to improve the manuscript. The manuscript has been substantially refined and improved according to the reviewers' comments. The authors hope this revision has improved the manuscript to the reviewers' and editor's satisfaction level.

Reviewer(s)' Comments to Author:

Reviewer: 1

The comments suggested by reviewer are not related to this paper

Reviewer: 2

Comments:

1. In the abstract section, I have noticed some part that need to be improved to better conform to the writing norms of academic paper abstract.

Thank you for the suggestions. We have revised the abstract according to the expert's feedback and with reference to the journal's format. Please see the updated abstract section for details.

The introduction section of this article lacks logic. This part should mainly include research background, research purpose, literature review, research content and research contribution. But this part does not make the above content clear to the reader.

Thank you for the expert's suggestions. After careful consideration, the authors have completely revised the introduction section of this paper. The specific logical framework for the revisions is as follows:

1. First Paragraph: It begins with an overview of the current state and trends of the global industrial revolution, linking it to China's macro policy environment for intelligent transformation and the development and application of industrial robotics. Against this broader context, the research subject of this paper—artificial intelligence (AI)—is introduced. This establishes the significant social and economic value of studying the effects of AI applications.

2. Second Paragraph: The theoretical basis is laid out, focusing on how the integration of AI and manufacturing can drive high-quality development in export trade. It identifies export technological complexity as a representative metric for evaluating the high-quality development of manufacturing exports. Additionally, it highlights the growing issue of China's export trade being large in scale but lacking in competitiveness. This serves to introduce the core research question and objective of the paper: how AI can empower the enhancement of export technological complexity and promote the high-quality development of China's manufacturing exports.

3. Third, Fourth, and Fifth Paragraphs: These sections provide a literature review. Respectively, they cover the research on the effects of AI applications, the effects of AI applications in the field of international trade, and the effects of AI applications specifically on export technological complexity. Each paragraph acts as a bridge between the preceding and subsequent sections, presenting a logical progression of theoretical developments. Through this structured review, the lack of existing research on the effects of AI on export technological complexity is identified. This reinforces the practical and theoretical significance of the research question and objective, lending the study scientific validity and relevance.

4. Sixth Paragraph: This summarizes the marginal contributions (innovative points) of the research, serving as a unifying thread throughout the paper.

This revised introduction ensures a clearer logical flow and emphasizes the significance of the study.

3. Contributions: Although some contributions are listed, but these contributions have been confirmed by a large number of articles, I don't think they belong to the real innovation. I suggest that the authors carefully summarize and refine the innovation points of this study and put forward 2-3 contributions that belong to this paper. In particular, I suggest a comparative analysis with relevant published articles.

Thank you for the suggestions. After careful consideration, the authors have summarized the marginal contributions (innovative points) of this paper into the following three aspects:

1. Research Perspective and Methodology:

While most existing literature evaluates export technological complexity from macro perspectives, such as industry or regional levels, this paper expands the research to the micro-level of individual enterprises. Additionally, it fully considers the potential impact of variations in export product quality when constructing measures of export technological complexity. This effectively addresses the issue of overestimating a country’s or region’s overall export technological complexity level when using HS6-digit product classification. By adopting this research perspective and methodology, the study not only reveals deeper insights into the evolution of export technological complexity within Chinese manufacturing enterprises but also broadens the research scope in the intersection of AI and international trade, offering new pathways and perspectives for subsequent studies in this field.

2. Exploration of Heterogeneity:

This paper goes beyond examining the general effects of AI applications to focus on their differentiated impacts under various micro (ownership structure of enterprises), meso (industry characteristics), and macro (regional disparities) contexts. By providing extensive empirical evidence, this study offers robust support for understanding the specific roles of AI in different types of enterprises, enriching the discourse on the diverse implications of AI applications.

3. Mechanism Exploration:

The study delves into the internal mechanisms through which AI influences enterprise export technological complexity, focusing on two core dimensions: optimizing enterprise labor structures and enhancing their innovation capabilities. This systematic exploration not only enriches the theoretical framework of AI’s effects on export trade but also provides a solid theoretical foundation for future related research.

For further details, please refer to the literature review section.

Literature review: There is no literature review in this paper. I suggest the authors to make a literature summary in the first part of the introduction, analyze the research content of existing literature, and propose the research content of this paper. Finally, this article cites literature that is too old and should cite literature from the last three years. These documents may be helpful for your revision, you can refer to or cite: Green Finance: The Catalyst for Artificial Intelligence and Energy Efficiency in Chinese Urban Sustainable Development. Energy Economics; Is Artificial Intelligence A Curse or A Blessing For Enterprise Energy Intensity? Evidence from China. Energy Economics; Breaking Boundaries: The Influence of Social Insurance Collection System Reform on Corporate Innovation Strategies. Applied Economics.

Thank you for the expert's suggestions. After careful consideration, the authors have conducted a literature review in the first section (Introduction) of the paper. The review is divided into three main aspects:

1. Research on the Effects of AI Applications

2. Research on the Effects of AI Applications in the Field of International Trade

3. Research on the Effects of AI Applications Focusing on Export Technological Complexity

For the cited literature, the primary principle was to select highly relevant works that reflect the historical development of research in each aspect.

5. Data section: This section should clearly tell the reader what data to use and how to handle it.

Thank you for the expert's suggestions. We sincerely apologize for the confusion caused by the lack of clarity regarding the data sources. To address this, we have added details about the data sources used in this paper (see Line#).

Specifically, the data was obtained from the CSMAR database by Guotaian, the WIND database, and the China Industrial Statistics Yearbook, which include enterprise-level data. Using Excel, we applied VLOOKUP and other formulas to filter out ST and *ST listed companies, as well as samples with severe data deficiencies. This process resulted in a final dataset comprising a union of 6,960 sample observations from 870 publicly listed manufacturing enterprises.

Additionally, to mitigate the impact of outliers, we Winsorized the main continuous variables at the 1% level at both ends. Data analysis was conducted using Stata 15.0.

6.Results section: The results analysis does not provide an in-depth discussion of its economic implications, that is, the results are not discussed from an economic perspective. including a comparative analysis of the existing literature, specifically in the baseline regression results. This article should add some robustness tests.

Thank you for the expert's suggestions. We have addressed these issues one by one, as detailed below:

1. Revision of Research Conclusions and Policy Recommendations:

In line with the expert's suggestions, we revised the research conclusions and policy recommendations by integrating content from the empirical analysis section. Additionally, we included a discussion section prior to the conclusions to provide further context and insights. For details, please refer to the discussion section in the paper.

2. Modification of the Explanatory Variable:

Based on the expert's feedback and considerations of data availability, we standardized the explanatory variable for AI adoption. Instead of using Ln(machine book value/number of employees), we now represent it as Ln(machine book value). The robustness test results based on this change are presented in the table below.

Table 1: Robustness Test Results

Variable (1) (2)

Ln(ESI) Ln(ESI)

Ln(AI) 0.106***

(0.015)

Ln（book value of machinery） 0.087***

(0.012)

Ln(Size) 0.286*** 0.286***

(0.017) (0.017)

Ln(Tq) 0.112*** 0.112***

(0.033) (0.033)

Ln(ATR) 0.196*** 0.196***

(0.029) (0.029)

Ln(CFR) 0.493*** 0.493***

(0.104) (0.104)

Ln(Lev) 0.004 0.004

(0.067) (0.067)

Ln(OER) 0.699*** 0.699***

(0.126) (0.126)

Ln(ROA) -0.621*** -0.621***

(0.115) (0.115)

Ln(RGR) -0.017*** -0.017***

(0.005) (0.005)

Ln(FAR) -0.181* -0.180*

(0.092) (0.092)

Ln(MER) -1.652*** -1.652***

(0.185) (0.185)

Ln(EI) 0.071*** 0.071***

(0.008) (0.008)

Ln(Age) 0.022*** 0.022***

(0.004) (0.004)

_cons 2.596*** 2.598***

(0.378) (0.378)

N 6960 6960

Industry fixed effects Yes Yes

Year fixed effect Yes Yes

R2 0.1893 0.1967

From Table 1, it can be observed that after replacing the core explanatory variable, the level of AI development remains significant at the 1% level, with a positive coefficient. This result is consistent with the baseline regression model. Both measures of AI development indicate a significant positive impact on the export technological complexity of enterprises, providing strong evidence for the robustness of the model used in this study.

7. Format specification: I recommend the authors to check the full text carefully. At present, the full text has many formatting errors, such as tables, literature citations and so on.

Thank you for the expert's suggestions. We have thoroughly reviewed and revised the language throughout the paper. Additionally, we have made adjustments to the tables, citations, and references. Please refer to the full text for the specific changes.。

Referee: 3

1. The paper is poorly written and there is a lot of Chinglish. The authors should polish the language and make the paper readable.

2. The literature review part in the introduction is messy. Also, the review is not in the correct format. For example,” Gregory et al. believe that using…” should be “Gregory et al. (2016) believe that using…”,” which it does not have an advantage.[13]” should be” which it does not have an advantage [13].”.

Thank you for the expert's suggestion. In this paper, the third, fourth, and fifth paragraphs of the introduction section have been revised to reorganize the literature review. These sections respectively provide:

1. A review of research on the effects of AI applications,

2. A review of research focusing on the effects of AI applications in the field of international trade, and

3. A review of research concentrating on the effects of AI applications on export technological complexity.

Through logical progression and detailed analysis, these reviews lead to the research questions and objectives of this paper. The format of the literature review has been adjusted to align with the review format of PLOS ONE (e.g., Hausmann et al., 2007; Goldfarb and Trefler, 2018).

Write all symbols with special meanings (such as j, t, N, P...) in the text using a formula editor.

Thank you for the expert's suggestion. As the expert rightly pointed out, the equations in the methodology section were not formatted using a formula editor. The authors have now re-edited all equations and symbols with special meanings using a formula editor for clarity and accuracy.

3. Please unify the format of Hypothesis H1, Hypothesis H2, and Hypothesis H3.

As the expert rightly pointed out, in the previous version, we noticed an inconsistency in the formatting of Hypothesis H2 compared to the other two hypotheses (H1 and H3). Specifically, H2 was not italicized, unlike the others. We deeply regret any inconvenience this oversight may have caused readers in understanding the text.

To ensure consistency and professionalism in the article, we have made the suggested adjustment and reformatted Hypothesis H2 to italicize it, aligning it with the formatting of the other hypotheses. This revision not only enhances the overall aesthetic quality of the article but also helps readers clearly differentiate and identify each hypothesis.

4. Where is Figure 2-1?

In the original text, we referred to "Figure 2-1" in the hypothesis testing section. However, due to an oversight, the corresponding figure was incorrectly labeled as "Figure 1." This inconsistency may have caused confusion for readers and affected the coherence and readability of the article, for which we sincerely apologize.

To resolve this issue, we have revised the text and corrected the reference to "Figure 2-1," aligning it with the actual label "Figure 1" in the figure. This adjustment ensures the accuracy of the relationship between the text and the figure, helping readers better understand and follow the information presented in the article.

5. What is the "Cathay Pacific listed company" mentioned in the introduction? The article did not mention any relationship with the data analysis in the following text.

Thank you very much for your detailed review of my article and for pointing out the issue regarding the "Cathay Pacific listed company" mentioned in the introduction. Upon careful reflection, we realize that the phrasing in the article may indeed have caused confusion for readers, and we sincerely apologize for this oversight.

The "Cathay Pacific listed company" mentioned in the text actually refers to one of the data sources used for our analysis, specifically the CSMAR database provided by Guotaian. We have clarified this in the revised text to ensure accurate understanding.

6. In the abstract, the data used is from 2008 to 2021, and in page 19, the investigation period is from 2014 to 2021. And why not study until 2022 or 2023?

Thank you very much for your detailed review of my article and for highlighting the inconsistency regarding the data time range as well as the question about the exclusion of 2022 and 2023 data. After a thorough review, we have provided a detailed explanation and clarification.

In the abstract, the data time range is mentioned as 2008 to 2021. This was intended to provide a broader historical context to demonstrate the overall trends of the research topic during this period. However, in the main text on page 19, we specifically describe the investigation and analysis period as 2014 to 2021. This adjustment in the time frame was made based on considerations of data availability and completeness.

Regarding the exclusion of 2022 and 2023 data, we conducted an in-depth discussion. Unfortunately, despite our efforts to collect the most recent data, we found that data from 2022 and 2023 were not sufficiently comprehensive, particularly for the specific indicators and data points critical to our study. Due to the scarcity and incompleteness of this data, we were concerned th

Attachment Submitted filename: Response to Reviwer12.10(1)(2).docx

# Peer review: Decision Letter 1

**DOI:** 10.1371/journal.pone.0351061.r003

**Authors:** Van Thanh Tien Nguyen, Van Thanh Tien Nguyen

5 Feb 2025

PONE-D-24-35896R1

Research on the Impact of Artificial Intelligence on the Export Technological Complexity of Chinese Manufacturing Enterprises: An Analysis Based on Mediating Effects

PLOS ONE

Dear Dr. Li,

Thank you for submitting your manuscript to PLOS ONE. After careful consideration, we feel that it has merit but does not fully meet PLOS ONE’s publication criteria as it currently stands. Therefore, we invite you to submit a revised version of the manuscript that addresses the points raised during the review process.

Please submit your revised manuscript by Mar 22 2025 11:59PM. If you will need more time than this to complete your revisions, please reply to this message or contact the journal office at plosone@plos.org. When you're ready to submit your revision, log on to https://www.editorialmanager.com/pone/ and select the 'Submissions Needing Revision' folder to locate your manuscript file.

Please include the following items when submitting your revised manuscript:

- A rebuttal letter that responds to each point raised by the academic editor and reviewer(s). You should upload this letter as a separate file labeled 'Response to Reviewers'.
- A marked-up copy of your manuscript that highlights changes made to the original version. You should upload this as a separate file labeled 'Revised Manuscript with Track Changes'.
- An unmarked version of your revised paper without tracked changes. You should upload this as a separate file labeled 'Manuscript'.

-->If you would like to make changes to your financial disclosure, please include your updated statement in your cover letter. Guidelines for resubmitting your figure files are available below the reviewer comments at the end of this letter.

If applicable, we recommend that you deposit your laboratory protocols in protocols.io to enhance the reproducibility of your results. Protocols.io assigns your protocol its own identifier (DOI) so that it can be cited independently in the future. For instructions see: https://journals.plos.org/plosone/s/submission-guidelines#loc-laboratory-protocols. Additionally, PLOS ONE offers an option for publishing peer-reviewed Lab Protocol articles, which describe protocols hosted on protocols.io. Read more information on sharing protocols at https://plos.org/protocols?utm_medium=editorial-email&utm_source=authorletters&utm_campaign=protocols.

We look forward to receiving your revised manuscript.

Kind regards,

Van Thanh Tien Nguyen, Ph.D.

Academic Editor

PLOS ONE

Journal Requirements:

Please review your reference list to ensure that it is complete and correct. If you have cited papers that have been retracted, please include the rationale for doing so in the manuscript text, or remove these references and replace them with relevant current references. Any changes to the reference list should be mentioned in the rebuttal letter that accompanies your revised manuscript. If you need to cite a retracted article, indicate the article’s retracted status in the References list and also include a citation and full reference for the retraction notice.

Additional Editor Comments:

Please revise the manuscript following the reviewer's suggestion. Thank you.

[Note: HTML markup is below. Please do not edit.]

Reviewers' comments:

Reviewer's Responses to Questions

**Comments to the Author**

1. If the authors have adequately addressed your comments raised in a previous round of review and you feel that this manuscript is now acceptable for publication, you may indicate that here to bypass the “Comments to the Author” section, enter your conflict of interest statement in the “Confidential to Editor” section, and submit your "Accept" recommendation.

Reviewer #1: All comments have been addressed

Reviewer #2: All comments have been addressed

**********

2. Is the manuscript technically sound, and do the data support the conclusions?

The manuscript must describe a technically sound piece of scientific research with data that supports the conclusions. Experiments must have been conducted rigorously, with appropriate controls, replication, and sample sizes. The conclusions must be drawn appropriately based on the data presented.

Reviewer #1: Yes

Reviewer #2: Yes

**********

3. Has the statistical analysis been performed appropriately and rigorously?

Reviewer #1: Yes

Reviewer #2: Yes

**********

4. Have the authors made all data underlying the findings in their manuscript fully available?

The PLOS Data policy requires authors to make all data underlying the findings described in their manuscript fully available without restriction, with rare exception (please refer to the Data Availability Statement in the manuscript PDF file). The data should be provided as part of the manuscript or its supporting information, or deposited to a public repository. For example, in addition to summary statistics, the data points behind means, medians and variance measures should be available. If there are restrictions on publicly sharing data—e.g. participant privacy or use of data from a third party—those must be specified.

Reviewer #1: Yes

Reviewer #2: Yes

**********

5. Is the manuscript presented in an intelligible fashion and written in standard English?

PLOS ONE does not copyedit accepted manuscripts, so the language in submitted articles must be clear, correct, and unambiguous. Any typographical or grammatical errors should be corrected at revision, so please note any specific errors here.

Reviewer #1: Yes

Reviewer #2: Yes

**********

6. Review Comments to the Author

Please use the space provided to explain your answers to the questions above. You may also include additional comments for the author, including concerns about dual publication, research ethics, or publication ethics. (Please upload your review as an attachment if it exceeds 20,000 characters)

Reviewer #1: This paper examines the impact and mechanism of AI on the export technological complexity of Chinese manufacturing enterprises from a corporate perspective. It utilizes data from listed manufacturing companies on the Shanghai and Shenzhen A-shares from 2008 to 2021 and employs a fixed-effects model. After review, it can be seen that the author has made some modifications according to the previous suggestions, but there are still following issues:

1. In 2.2.3 section, the author elaborates on the impact of AI on the export technological complexity of manufacturing enterprises by enhancing innovation capability, and analyzes the influence of AI on the innovation from three aspects. It is suggested to organize these three points into paragraph and use conjunctions such as "firstly", "secondly", and "thirdly".

2. In all regression result tables, the significance of the data is not indicated by an asterisk (*). The capitalization of "yes" and "Yes" in "Table 4-1 Baseline regression result" is not consistent, and there is a lack of relevant explanation for significance identification in the Note.

3. The regression results of the mediation mechanism tests in "Table 4-7" and "Table 4-8" did not control industry and year fixed effects.

4. In“4. Empirical Analysis”section, it is suggested to place "4.3.4 Mechanism Testing" after "4.1 Regression Results".

5. Pay attention to standardizing the article format, indent the first line of all paragraphs, and adjust the font of the "5. Discussion" section.

Overall, the format and language expression of this article need to be further improved.

Reviewer #2: (No Response)

**********

7. PLOS authors have the option to publish the peer review history of their article (what does this mean?). If published, this will include your full peer review and any attached files.

If you choose “no”, your identity will remain anonymous but your review may still be made public.

**Do you want your identity to be public for this peer review?**  For information about this choice, including consent withdrawal, please see our Privacy Policy.

Reviewer #1: No

Reviewer #2: No

**********

[NOTE: If reviewer comments were submitted as an attachment file, they will be attached to this email and accessible via the submission site. Please log into your account, locate the manuscript record, and check for the action link "View Attachments". If this link does not appear, there are no attachment files.]

While revising your submission, please upload your figure files to the Preflight Analysis and Conversion Engine (PACE) digital diagnostic tool, https://pacev2.apexcovantage.com/. PACE helps ensure that figures meet PLOS requirements. To use PACE, you must first register as a user. Registration is free. Then, login and navigate to the UPLOAD tab, where you will find detailed instructions on how to use the tool. If you encounter any issues or have any questions when using PACE, please email PLOS at figures@plos.org. Please note that Supporting Information files do not need this step.

Attachment Submitted filename: Comments.docx

# Author comment: Author response to Decision Letter 2

**DOI:** 10.1371/journal.pone.0351061.r004

22 Mar 2025

Our Response

Dear Reviewers:

The authors are thankful to the editor and reviewers for their in-depth review and input to improve the manuscript. The manuscript has been substantially refined and improved according to the reviewers' comments. The authors hope this revision has improved the manuscript to the reviewers' and editor's satisfaction level.

Reviewer(s)' Comments to Author:

Referee: 2

Comments:

1. In 2.2.3 section, the author elaborates on the impact of AI on the export technological complexity of manufacturing enterprises by enhancing innovation capability, and analyzes the influence of AI on the innovation from three aspects. It is suggested to organize these three points into paragraph and use conjunctions such as "firstly", "secondly", and "thirdly".

[Answer] Thank you for the expert's suggestions. We have revised Section 2.2.3 according to the expert's comments. Please see the updated Section 2.2.3 for details.

2. In all regression result tables, the significance of the data is not indicated by an asterisk (*). The capitalization of "yes" and "Yes" in "Table 4-1 Baseline regression result" is not consistent, and there is a lack of relevant explanation for significance identification in the Note.[Answer] Thank you for the expert's suggestions.

[Answer] Thank you for the expert's suggestions. Due to our oversight, we have caused misunderstanding for the expert and readers, and we sincerely apologize. Following the expert's suggestions, we have added asterisks () to all regression results and included explanatory notes below each regression result table. Additionally, we have standardized the capitalization of "Yes" in all regression results. Please see the revised regression result tables throughout the manuscript for details.

3. The regression results of the mediation mechanism tests in "Table 4-7" and "Table 4-8" did not control industry and year fixed effects.

[Answer] Thank you for the expert's suggestions. Due to the authors' oversight, we have caused confusion for the expert, and we deeply apologize. Based on the expert's suggestions, we have added controls for industry and year fixed effects to the tables.

4.In“4. Empirical Analysis”section, it is suggested to place "4.3.4 Mechanism Testing" after "4.1 Regression Results".

[Answer] Thank you for the expert's suggestions. Following the expert's advice, the authors have moved "4.3.4 Mechanism Testing" to follow "4.1 Regression Results," renumbered it as "4.2," and adjusted the subsequent sections, headings, and table numbers accordingly. Please see the revised fourth section of the article for details.

5. Pay attention to standardizing the article format, indent the first line of all paragraphs, and adjust the font of the "5. Discussion" section.

[Answer] Thank you for the expert's suggestions. The authors have adjusted the formatting throughout the manuscript, particularly ensuring that the first line of all paragraphs is indented. Additionally, the font in the Discussion section has been revised. Please see the full manuscript and the Discussion section for details.

6. Overall, the format and language expression of this article need to be further improved.

[Answer] Thank you for the expert's suggestions. We apologize for the confusion caused by the formatting and language expression issues. Based on the expert's suggestions, we have revised the language and formatting throughout the manuscript.

Attachment Submitted filename: Response to Reviwer2025.02.20.docx

# Peer review: Decision Letter 2

**DOI:** 10.1371/journal.pone.0351061.r005

**Authors:** Van Thanh Tien Nguyen, Van Thanh Tien Nguyen, Van Thanh Tien Nguyen

9 Jun 2025

PONE-D-24-35896R2

Research on the Impact of Artificial Intelligence on the Export Technological Complexity of Chinese Manufacturing Enterprises: An Analysis Based on Mediating Effects

PLOS ONE

Dear Dr. Li,

Thank you for submitting your manuscript to PLOS ONE. After careful consideration, we feel that it has merit but does not fully meet PLOS ONE’s publication criteria as it currently stands. Therefore, we invite you to submit a revised version of the manuscript that addresses the points raised during the review process.

Please submit your revised manuscript by Jul 24 2025 11:59PM. If you will need more time than this to complete your revisions, please reply to this message or contact the journal office at plosone@plos.org. When you're ready to submit your revision, log on to https://www.editorialmanager.com/pone/ and select the 'Submissions Needing Revision' folder to locate your manuscript file.

Please include the following items when submitting your revised manuscript:

- A rebuttal letter that responds to each point raised by the academic editor and reviewer(s). You should upload this letter as a separate file labeled 'Response to Reviewers'.
- A marked-up copy of your manuscript that highlights changes made to the original version. You should upload this as a separate file labeled 'Revised Manuscript with Track Changes'.
- An unmarked version of your revised paper without tracked changes. You should upload this as a separate file labeled 'Manuscript'.

-->If you would like to make changes to your financial disclosure, please include your updated statement in your cover letter. Guidelines for resubmitting your figure files are available below the reviewer comments at the end of this letter.

If applicable, we recommend that you deposit your laboratory protocols in protocols.io to enhance the reproducibility of your results. Protocols.io assigns your protocol its own identifier (DOI) so that it can be cited independently in the future. For instructions see: https://journals.plos.org/plosone/s/submission-guidelines#loc-laboratory-protocols. Additionally, PLOS ONE offers an option for publishing peer-reviewed Lab Protocol articles, which describe protocols hosted on protocols.io. Read more information on sharing protocols at https://plos.org/protocols?utm_medium=editorial-email&utm_source=authorletters&utm_campaign=protocols.

We look forward to receiving your revised manuscript.

Kind regards,

Van Thanh Tien Nguyen, Ph.D.

Academic Editor

PLOS ONE

Journal Requirements:

Please review your reference list to ensure that it is complete and correct. If you have cited papers that have been retracted, please include the rationale for doing so in the manuscript text, or remove these references and replace them with relevant current references. Any changes to the reference list should be mentioned in the rebuttal letter that accompanies your revised manuscript. If you need to cite a retracted article, indicate the article’s retracted status in the References list and also include a citation and full reference for the retraction notice.

Additional Editor Comments:

Please note that at least one reviewer has suggested citing specific references. Kindly check the relevance of these suggested references carefully. Please remember that citing these references is not mandatory for PLOS ONE.

[Note: HTML markup is below. Please do not edit.]

Reviewers' comments:

Reviewer's Responses to Questions

**Comments to the Author**

1. If the authors have adequately addressed your comments raised in a previous round of review and you feel that this manuscript is now acceptable for publication, you may indicate that here to bypass the “Comments to the Author” section, enter your conflict of interest statement in the “Confidential to Editor” section, and submit your "Accept" recommendation.

Reviewer #1: All comments have been addressed

Reviewer #4: (No Response)

**********

2. Is the manuscript technically sound, and do the data support the conclusions?

The manuscript must describe a technically sound piece of scientific research with data that supports the conclusions. Experiments must have been conducted rigorously, with appropriate controls, replication, and sample sizes. The conclusions must be drawn appropriately based on the data presented.

Reviewer #1: Yes

Reviewer #4: (No Response)

**********

3. Has the statistical analysis been performed appropriately and rigorously?

Reviewer #1: Yes

Reviewer #4: (No Response)

**********

4. Have the authors made all data underlying the findings in their manuscript fully available?

The PLOS Data policy requires authors to make all data underlying the findings described in their manuscript fully available without restriction, with rare exception (please refer to the Data Availability Statement in the manuscript PDF file). The data should be provided as part of the manuscript or its supporting information, or deposited to a public repository. For example, in addition to summary statistics, the data points behind means, medians and variance measures should be available. If there are restrictions on publicly sharing data—e.g. participant privacy or use of data from a third party—those must be specified.

Reviewer #1: Yes

Reviewer #4: (No Response)

**********

5. Is the manuscript presented in an intelligible fashion and written in standard English?

PLOS ONE does not copyedit accepted manuscripts, so the language in submitted articles must be clear, correct, and unambiguous. Any typographical or grammatical errors should be corrected at revision, so please note any specific errors here.

Reviewer #1: Yes

Reviewer #4: (No Response)

**********

6. Review Comments to the Author

Please use the space provided to explain your answers to the questions above. You may also include additional comments for the author, including concerns about dual publication, research ethics, or publication ethics. (Please upload your review as an attachment if it exceeds 20,000 characters)

Reviewer #1: The topic of the paper has good theoretical and practical value, focusing on the impact of artificial intelligence (AI) on the export technology complexity of Chinese manufacturing enterprises. The research framework is complete and the empirical analysis is detailed. However, the paper has the following questionable issues:

1.The introduction of the research question in the paper is a bit sudden; Insufficient literature review on the economic effects of AI (such as neglecting literature on AI and growth like Aghion, and lacking relevant research on China).

2. Figure 1 on page 13 is labeled as Figure 2-1 in the main text, which is incorrect.

3. The rationality of AI's proxy variables is insufficient. The paper uses "per capita book value of mechanical equipment (Ln (book value of mechanical equipment/number of employees)" as a proxy variable for AI, but fails to distinguish between traditional machinery and AI technologies such as algorithms and deep learning. It is recommended to use indicators such as the number of enterprise AI patents, the proportion of AI related R&D investment, or industry level robot penetration rate (IFR data) as measurement indicators, which may be more effective.

4. Additional explanation is needed for the calculation of export technology complexity (ESI). The weighting method for enterprise level ESI in formula (12) is reasonable, but it does not explain how to handle the matching problem between product classification (HS 6-digit code) and enterprise products. Suggestion: Supplement the details of the data matching process (such as the docking method between customs data and enterprise export products) to avoid selective bias.

5.The policy recommendations lack specificity, and the correlation between policy recommendations and empirical findings should be strengthened.

6. The reference format is inconsistent. Some literature lacks page numbers (such as [24]), and some are cited repeatedly (such as [13] and [14] both by Dixit & Stiglitz, 1977).

Reviewer #4: This paper examines the impact and mechanism of AI on the export technological complexity of Chinese manufacturing enterprises from a corporate perspective. It finds that AI indirectly enhances the export technology complexity of manufacturing firms by optimizing labor structure and improving innovation capabilities. The study holds significant theoretical and practical value. However, before submission, the following minor revisions are recommended:

1. In the introduction, please clearly articulate the contributions of this research.

2. Key literature should be incorporated into the literature review, such as:

DOI: 10.1109/TEM.2025.3543210

doi.org/10.1016/j.jebo.2023.05.008

doi.org/10.1016/j.jfineco.2023.103745

3. Why are firm fixed effects not included in the main regression model? Controlling for firm fixed effects could enhance the model's explanatory power.

4. Please carefully proofread and check for grammatical issues; further language polishing is necessary. References 12 and 13 are duplicates.

**********

7. PLOS authors have the option to publish the peer review history of their article (what does this mean?). If published, this will include your full peer review and any attached files.

If you choose “no”, your identity will remain anonymous but your review may still be made public.

**Do you want your identity to be public for this peer review?**  For information about this choice, including consent withdrawal, please see our Privacy Policy.

Reviewer #1: No

Reviewer #4: No

**********

[NOTE: If reviewer comments were submitted as an attachment file, they will be attached to this email and accessible via the submission site. Please log into your account, locate the manuscript record, and check for the action link "View Attachments". If this link does not appear, there are no attachment files.]

While revising your submission, please upload your figure files to the Preflight Analysis and Conversion Engine (PACE) digital diagnostic tool, https://pacev2.apexcovantage.com/. PACE helps ensure that figures meet PLOS requirements. To use PACE, you must first register as a user. Registration is free. Then, login and navigate to the UPLOAD tab, where you will find detailed instructions on how to use the tool. If you encounter any issues or have any questions when using PACE, please email PLOS at figures@plos.org. Please note that Supporting Information files do not need this step.

# Author comment: Author response to Decision Letter 3

**DOI:** 10.1371/journal.pone.0351061.r006

1 Sep 2025

Our Response

Dear Reviewers:

The authors are thankful to the editor and reviewers for their in-depth review and input to improve the manuscript. The manuscript has been substantially refined and improved according to the reviewers' comments. The authors hope this revision has improved the manuscript to the reviewers' and editor's satisfaction level.

Reviewer(s)' Comments to Author:

Referee: 1

Comments:

1.The introduction of the research question in the paper is a bit sudden; Insufficient literature review on the economic effects of AI (such as neglecting literature on AI and growth like Aghion, and lacking relevant research on China).

Response: Thank you for the suggestion. We have revised the Introduction section to include the research question and a clear research objective at the end (see p. 6). In the second paragraph of the Introduction, foundational theories by Aghion and empirical studies by Chinese scholars have been added to strengthen the theoretical framework on AI’s economic effects (see pp. 4–5). Additionally, the literature gap section has been supplemented to address the "macro-micro linkage gaps" (see p. 5).

2. Figure 1 on page 13 is labeled as Figure 2-1 in the main text, which is incorrect.

Response: Thank you for the expert’s suggestions. We have corrected the figure numbering from Figure 2-1 to Figure 1 as advised (see p. 14).

3. The rationality of AI's proxy variables is insufficient. The paper uses "per capita book value of mechanical equipment (Ln (book value of mechanical equipment/number of employees)" as a proxy variable for AI, but fails to distinguish between traditional machinery and AI technologies such as algorithms and deep learning. It is recommended to use indicators such as the number of enterprise AI patents, the proportion of AI related R&D investment, or industry level robot penetration rate (IFR data) as measurement indicators, which may be more effective.

Response: Thank you for the expert’s suggestions. The indicators recommended—such as the number of enterprise AI patents, the proportion of AI-related R&D investment, and the industry-level robot penetration rate (IFR data)—are theoretically more reasonable and effective for measuring enterprise AI development levels. However, practical challenges exist. Regarding AI patents, there is no comprehensive international disclosure mechanism, and many enterprises do not publicly report such data due to commercial confidentiality or inconsistent data management, making it difficult to obtain high-quality firm-level information. For AI-related R&D investment, inconsistent classification standards and the lack of separate accounting in some firms hinder accurate measurement. The industry-level robot penetration rate (IFR data) reflects only national or industry-wide trends and cannot capture micro-level differences among individual enterprises. Given these limitations and data availability constraints, this study uses the per capita book value of mechanical equipment (Ln [book value of mechanical equipment/number of employees]) as a proxy variable. Although it cannot fully distinguish between traditional machinery and AI technologies, it partially reflects enterprise investment in mechanical equipment, which relates to technological upgrades and AI applications. Future research will explore more suitable indicators as data conditions improve.

4. Additional explanation is needed for the calculation of export technology complexity (ESI). The weighting method for enterprise level ESI in formula (12) is reasonable, but it does not explain how to handle the matching problem between product classification (HS 6-digit code) and enterprise products. Suggestion: Supplement the details of the data matching process (such as the docking method between customs data and enterprise export products) to avoid selective bias.

Response: Thank you for the expert’s suggestions. A detailed explanation of the data matching process has been added to the last paragraph of Section 3.2.1 (see p. 16).

5.The policy recommendations lack specificity, and the correlation between policy recommendations and empirical findings should be strengthened.

Response: Thank you for the expert’s suggestions. The policy recommendations section has been revised accordingly (see pp. 32–34).

6. The reference format is inconsistent. Some literature lacks page numbers (such as [24]), and some are cited repeatedly (such as [13] and [14] both by Dixit & Stiglitz, 1977).

Response: Thank you for the expert’s suggestions. Upon review, we identified errors in the literature citations. The original reference [24] has been updated to [30] after reordering. Duplicate citations [13] and [14] have been removed, and all references have been renumbered accordingly throughout the paper.

Referee: 4

Comments:

1. In the introduction, please clearly articulate the contributions of this research.

Response: Thank you for the expert’s suggestions. The contributions of this research have been clearly articulated in the Introduction section (see p. 6).

2. Key literature should be incorporated into the literature review, such as:

DOI: 10.1109/TEM.2025.3543210

doi.org/10.1016/j.jebo.2023.05.008

doi.org/10.1016/j.jfineco.2023.103745

Response: Thank you for the expert’s suggestions. Key literature has been added to the literature review section (see pp. 5–6), and the references have been updated accordingly.

3. Why are firm fixed effects not included in the main regression model? Controlling for firm fixed effects could enhance the model's explanatory power.

Response: Thank you for the expert’s suggestions. The main regression model does not include firm fixed effects for the following reasons:

Sample enterprises are grouped into three major industry categories based on their characteristics, and industry fixed effects are included in the model.

Theoretically, while firm fixed effects control for time-invariant firm-level traits (e.g., corporate culture, management style), these are not the primary determinants of the relationship studied here. Industry-level factors such as technological development, market competition, and policy environment more directly influence key outcomes like export technology complexity. Controlling for industry fixed effects better captures industry heterogeneity and common shocks, allowing clearer identification of the core explanatory variables’ effects.

Empirically, including firm fixed effects would greatly reduce degrees of freedom given the large sample size and time span, risking multicollinearity and less stable estimates. Industry fixed effects offer a better balance between degrees of freedom, precision, and explanatory power.

Future research with larger samples and improved data structures may explore adding both firm and industry fixed effects to enhance rigor and control for more factors comprehensively.

4. Please carefully proofread and check for grammatical issues; further language polishing is necessary. References 12 and 13 are duplicates.

Response: Thank you for the experts’ suggestions. We have proofread the paper and duplicate reference has been removed.

7. PLOS authors have the option to publish the peer review history of their article (what does this mean?). If published, this will include your full peer review and any attached files.

Response: This means that when authors publish their paper with PLOS journals, they can choose to make the entire peer review process publicly available. This includes all the reviewer comments, author responses, editorial decisions, and any files exchanged during review. It adds transparency by letting readers see the discussion and revisions behind the final published article. We agree with the expert's recommendation.

Attachment Submitted filename: v3_Response to Reviwer(2).docx

# Peer review: Decision Letter 3

**DOI:** 10.1371/journal.pone.0351061.r007

**Authors:** Van Thanh Tien Nguyen, Van Thanh Tien Nguyen, Van Thanh Tien Nguyen, Van Thanh Tien Nguyen

12 Nov 2025

PONE-D-24-35896R3

Research on the Impact of Artificial Intelligence on the Export Technological Complexity of Chinese Manufacturing Enterprises: An Analysis Based on Mediating Effects

PLOS ONE

Dear Dr. Li,

Thank you for submitting your manuscript to PLOS ONE. After careful consideration, we feel that it has merit but does not fully meet PLOS ONE’s publication criteria as it currently stands. Therefore, we invite you to submit a revised version of the manuscript that addresses the points raised during the review process.

Please submit your revised manuscript by Dec 27 2025 11:59PM. If you will need more time than this to complete your revisions, please reply to this message or contact the journal office at plosone@plos.org. When you're ready to submit your revision, log on to https://www.editorialmanager.com/pone/ and select the 'Submissions Needing Revision' folder to locate your manuscript file.

Please include the following items when submitting your revised manuscript:

- A rebuttal letter that responds to each point raised by the academic editor and reviewer(s). You should upload this letter as a separate file labeled 'Response to Reviewers'.
- A marked-up copy of your manuscript that highlights changes made to the original version. You should upload this as a separate file labeled 'Revised Manuscript with Track Changes'.
- An unmarked version of your revised paper without tracked changes. You should upload this as a separate file labeled 'Manuscript'.

-->If you would like to make changes to your financial disclosure, please include your updated statement in your cover letter. Guidelines for resubmitting your figure files are available below the reviewer comments at the end of this letter.

If applicable, we recommend that you deposit your laboratory protocols in protocols.io to enhance the reproducibility of your results. Protocols.io assigns your protocol its own identifier (DOI) so that it can be cited independently in the future. For instructions see: https://journals.plos.org/plosone/s/submission-guidelines#loc-laboratory-protocols. Additionally, PLOS ONE offers an option for publishing peer-reviewed Lab Protocol articles, which describe protocols hosted on protocols.io. Read more information on sharing protocols at https://plos.org/protocols?utm_medium=editorial-email&utm_source=authorletters&utm_campaign=protocols.

We look forward to receiving your revised manuscript.

Kind regards,

Van Thanh Tien Nguyen, Ph.D.

Academic Editor

PLOS ONE

Journal Requirements:

If the reviewer comments include a recommendation to cite specific previously published works, please review and evaluate these publications to determine whether they are relevant and should be cited. There is no requirement to cite these works unless the editor has indicated otherwise.

Please review your reference list to ensure that it is complete and correct. If you have cited papers that have been retracted, please include the rationale for doing so in the manuscript text, or remove these references and replace them with relevant current references. Any changes to the reference list should be mentioned in the rebuttal letter that accompanies your revised manuscript. If you need to cite a retracted article, indicate the article’s retracted status in the References list and also include a citation and full reference for the retraction notice.

Additional Editor Comments:

Dear Prof. Wenlong Li,

The manuscript is now in principle suitable for publication.

I thank you for your thorough revisions and response to the previous rounds of peer review. You have addressed the main concerns comprehensively, and the manuscript has been greatly improved.

Based on the final review, there are a few remaining minor adjustments required. Please treat this decision as a final technical check.

Specifically, please address the following points: "The policy recommendations lack specificity, and the correlation between policy recommendations and empirical findings should be strengthened."

These changes should be straightforward to implement. Upon resubmission, I will review the response to ensure these last points have been addressed, and I fully anticipate issuing an acceptance at that stage.

Thank you for submitting your excellent work to PLOS One.

Sincerely yours,

Dr. Van Thanh Tien Nguyen

Academic Editor

[Note: HTML markup is below. Please do not edit.]

Reviewers' comments:

Reviewer's Responses to Questions

**Comments to the Author**

1. If the authors have adequately addressed your comments raised in a previous round of review and you feel that this manuscript is now acceptable for publication, you may indicate that here to bypass the “Comments to the Author” section, enter your conflict of interest statement in the “Confidential to Editor” section, and submit your "Accept" recommendation.

Reviewer #1: All comments have been addressed

Reviewer #4: (No Response)

**********

2. Is the manuscript technically sound, and do the data support the conclusions?

The manuscript must describe a technically sound piece of scientific research with data that supports the conclusions. Experiments must have been conducted rigorously, with appropriate controls, replication, and sample sizes. The conclusions must be drawn appropriately based on the data presented.

Reviewer #1: Partly

Reviewer #4: (No Response)

**********

3. Has the statistical analysis been performed appropriately and rigorously?

Reviewer #1: Yes

Reviewer #4: (No Response)

**********

4. Have the authors made all data underlying the findings in their manuscript fully available?

The PLOS Data policy requires authors to make all data underlying the findings described in their manuscript fully available without restriction, with rare exception (please refer to the Data Availability Statement in the manuscript PDF file). The data should be provided as part of the manuscript or its supporting information, or deposited to a public repository. For example, in addition to summary statistics, the data points behind means, medians and variance measures should be available. If there are restrictions on publicly sharing data—e.g. participant privacy or use of data from a third party—those must be specified.

Reviewer #1: Yes

Reviewer #4: (No Response)

**********

5. Is the manuscript presented in an intelligible fashion and written in standard English?

PLOS ONE does not copyedit accepted manuscripts, so the language in submitted articles must be clear, correct, and unambiguous. Any typographical or grammatical errors should be corrected at revision, so please note any specific errors here.

Reviewer #1: Yes

Reviewer #4: (No Response)

**********

6. Review Comments to the Author

Please use the space provided to explain your answers to the questions above. You may also include additional comments for the author, including concerns about dual publication, research ethics, or publication ethics. (Please upload your review as an attachment if it exceeds 20,000 characters)

Reviewer #1: The topic of this paper, "Research on the Impact of Artificial Intelligence on the Technological Complexity of Chinese Manufacturing Enterprises' Exports: An Analysis Based on Mediation Effect," has important theoretical and practical significance. It attempts to explore the impact mechanism of artificial intelligence (AI) on export technological complexity from the micro enterprise level and conducts heterogeneity analysis. The overall framework is relatively complete. However, after careful review, the paper still has the following issues that are worth discussing and improving.

1.The literature review section lacks key references and demonstrates insufficient logical clarity. Specific manifestations include: (1) the review of studies on the economic effects of AI is not systematic, omitting foundational theoretical work such as that by Aghion et al. on AI and growth, as well as important empirical studies on AI and firm behavior in the Chinese context; (2) the review fails to clearly delineate the gaps in existing research and correspondingly highlight the marginal contributions of this study.

2.The depth of theoretical mechanism analysis is insufficient. Although two intermediary mechanisms, "labor structure optimization" and "innovation capability enhancement," have been proposed, the theoretical analysis section (Chapter 2) lacks in-depth elaboration on the micro mechanisms of how AI affects export technology complexity through these two paths, and lacks rigorous theoretical derivation and literature support.

3.The measurement of the core explanatory variable (AI) is not reasonable enough. The paper uses "per capita book value of mechanical equipment" as a proxy variable for AI, which has significant flaws. It cannot distinguish between traditional machinery and AI technology with algorithm and deep learning capabilities, making it difficult to accurately reflect the level of "artificial intelligence" of enterprises, which can easily lead to measurement errors and endogeneity problems. You can choose to use industry level robot penetration rate (IFR data) to match the industry in which the enterprise operates, or the number of AI related patent applications/authorizations for the enterprise. Text analysis can also be used to extract AI keyword frequency from annual reports as a measurement method.

4.The main regression model only controls for industry and year fixed effects, without controlling for firm fixed effects, and cannot exclude the influence of firm heterogeneity (such as corporate culture, management capabilities, etc.) that does not change over time on the estimation results.

5.The policy recommendations are too generalized and have weak correlation with empirical results. The suggestions put forward in the policy recommendations section are relatively vague (such as "strengthening AI technology research and development" and "optimizing labor structure"), and have not been closely integrated with the empirical findings of this article (such as heterogeneity results: state-owned enterprises and the eastern and central regions have more significant effects; intermediary mechanisms: the role of labor structure and innovation) to propose precise and actionable policy solutions.

Reviewer #4: The author has addressed my concerns. Congratulations on the author's hard work. I suggest publishing this paper.

**********

7. PLOS authors have the option to publish the peer review history of their article (what does this mean?). If published, this will include your full peer review and any attached files.

If you choose “no”, your identity will remain anonymous but your review may still be made public.

**Do you want your identity to be public for this peer review?**  For information about this choice, including consent withdrawal, please see our Privacy Policy.

Reviewer #1: No

Reviewer #4: No

**********

[NOTE: If reviewer comments were submitted as an attachment file, they will be attached to this email and accessible via the submission site. Please log into your account, locate the manuscript record, and check for the action link "View Attachments". If this link does not appear, there are no attachment files.]

To ensure your figures meet our technical requirements, please review our figure guidelines: https://journals.plos.org/plosone/s/figures

You may also use PLOS’s free figure tool, NAAS, to help you prepare publication quality figures: https://journals.plos.org/plosone/s/figures#loc-tools-for-figure-preparation.

NAAS will assess whether your figures meet our technical requirements by comparing each figure against our figure specifications.

# Author comment: Author response to Decision Letter 4

**DOI:** 10.1371/journal.pone.0351061.r008

15 Feb 2026

Research on the Impact of Artificial Intelligence on the Export Technological Complexity of Chinese Manufacturing Enterprises: An Analysis Based on Mediating Effects

Revision Notes

Dear Esteemed Reviewer：

Thank you very much for taking time out of your busy schedule to review our manuscript and for providing valuable revision suggestions, which have significantly contributed to improving the quality of our work. The authors have carefully examined the reviewer's comments, consulted additional literature, furthered our understanding, broken down the suggestions in detail, and revised the paper meticulously word by word.

Below, we address each of the reviewer's comments point by point:

Reviewer 1 Comments:

Comment 1:The literature review section lacks key references and suffers from insufficient logical clarity. Specific issues include: (1) The review of research on the economic effects of artificial intelligence is not systematic enough, omitting fundamental theoretical works, such as studies by Aghion et al. on AI and economic growth, as well as important empirical research on AI and firm behavior in the Chinese context; (2) The review fails to clearly delineate the gaps in existing research and correspondingly highlight the marginal contribution of this study.

Revision Explanation:

The following two modifications have been made to the literature review section:

（1）Key references have been added. Theoretical foundations now include studies by Aghion et al. (2017), Koch et al. (2021), and Brynjolfsson et al. (2019) on AI and economic growth and the labor market.

（2）The research gap and marginal contribution of the paper have been clearly articulated.

Summary of Revisions:

Please refer to the sections highlighted in red in the manuscript for your review.

Comment 2:The theoretical mechanism analysis is insufficiently deep. Although two mediating mechanisms, "labor structure optimization" and "innovation capability enhancement," are proposed, the theoretical analysis section (Chapter 2) lacks in-depth elaboration on the micro-mechanisms through which AI affects export technical sophistication via these two pathways. It also lacks rigorous theoretical derivation and literature support.

Revision Explanation:

The theoretical mechanisms section in Chapter 2 has been systematically deepened, with key additions of relevant literature including Yang Xiaoxia et al. (2024), Chen Jia et al. (2023), Zhao Chunming et al. (2025), and Jin Zehu et al. (2023). These provide a more solid theoretical basis and empirical support for the two transmission pathways of labor structure optimization and innovation capability enhancement. The revised mechanism analysis features more rigorous logic and more sufficientargumentation

Summary of Revisions:

Please refer to the sections highlighted in red in the manuscript for your review.

Comment 3:The measurement of the core explanatory variable (Artificial Intelligence AI) is not sufficiently reasonable. The paper uses "per capita book value of machinery and equipment" as a proxy variable for AI, which has significant defects. It fails to distinguish between traditional machinery and AI technologies involving algorithms and deep learning, thus making it difficult to accurately reflect the firm's "artificial intelligence" level. This is prone to measurement error and endogeneity issues. Alternatives include using industry-level robot penetration rates (IFR data) matched to the firm's industry, or using the number of AI-related patent applications/grants by firms. Additionally, text analysis methods can be employed to extract the frequency of AI keywords from annual reports as a measurement approach.

Revision Explanation:

Existing literature has used industry-level robot penetration rates (IFR data) matched to the industry of firms to measure AI levels.

Summary of Revisions:

Please refer to the sections highlighted in red in the manuscript for your review.

Comment 4:The main regression model only controls for industry and year fixed effects, without controlling for firm fixed effects. Consequently, it cannot rule out the influence of firm heterogeneity (e.g., corporate culture, management capabilities) on the estimation results.

Revision Explanation:

Heterogeneity analysis was conducted by dividing firms into state-owned and non-state-owned enterprises.

Summary of Revisions:

Please refer to the sections highlighted in red in the manuscript for your review.

Comment 5:The policy recommendations are too general and lack strong relevance to the empirical results. The suggestions in the policy recommendation section are relatively vague (e.g., "strengthen AI technology R&D," "optimize labor structure") and are not closely integrated with the empirical findings of this paper (e.g., heterogeneity results: more significant effects in state-owned enterprises and the eastern and central regions; mediating mechanisms: the roles of labor structure and innovation) to propose precise and actionable policy solutions.

Revision Explanation:

(I) Precise Measures Based on Benchmark Effects and Mediating Mechanisms

Policy Recommendations Targeting the "Labor Structure Optimization" Mediating Pathway:

Firm Level: Implement "AI Skills Upgrade Programs," collaborate with vocational schools and AI technology companies to provide targeted training on intelligent equipment operation, data processing, etc., for existing low-skilled employees; establish "High-Skilled Talent Incentive Mechanisms" to improve compensation and career advancement pathways for AI-related positions, attracting external high-skilled talent.

Government Level: Provide financial subsidies for AI skills training conducted by firms; build "AI Talent Training Bases" in manufacturing-intensive regions, focusing on cultivating practical skilled talent that meets enterprise needs; improve the vocational education system by adding majors such as Artificial Intelligence and Industrial Automation to alleviate the shortage of high-skilled talent.

Policy Recommendations Targeting the "Innovation Capability Enhancement" Mediating Pathway:

Firm Level: Increase AI R&D investment, focusing on AI technology R&D related to core products (e.g., intelligent production algorithms, AI systems for product quality inspection); strengthen collaboration with universities and research institutes to establish joint AI innovation laboratories, accelerating the commercialization of technological achievements.

Government Level: Establish a "Manufacturing AI Innovation Special Fund" to provide financial support for firms' AI R&D projects; optimize the intellectual property protection system, strengthening the protection of AI-related patents; build an "AI Technology Sharing Platform" to facilitate the transfer and diffusion of AI technological achievements from universities and research institutes to SMEs.

(II) Differentiated Policy Recommendations Based on Heterogeneity Results

Policy Recommendations Targeting State-Owned Enterprises (SOEs):

Leverage the exemplary and leading role of SOEs by supporting them in building "AI + Manufacturing" benchmark projects (e.g., smart factories, digital twin production lines) to provide replicable experiences for the industry; encourage SOEs to engage in mixed-ownership cooperation with private AI enterprises to avoid "reinventing the wheel" and enhance the efficiency of market-oriented application of AI technology.

Policy Recommendations Targeting Regional Disparities:

Eastern Region: Focus on building AI industry clusters, supporting cross-enterprise and cross-regional collaborative innovation in AI technology; promote the deep integration of AI with high-end manufacturing, focusing on high-tech export sectors such as semiconductors and precision machinery to further enhance export technical sophistication.

Central Region: Leverage the advantage of undertaking industrial relocation, focus on promoting mature AI application technologies (e.g., industrial robots, intelligent logistics systems) to accelerate the intelligent transformation of traditional manufacturing; strengthen technological cooperation with the eastern region to introduce advanced AI technologies and talents.

Western Region: Prioritize improving digital infrastructure (e.g., 5G networks, Industrial Internet) to lower the hardware threshold for AI application; conduct "AI Technology Popularization Training" to help enterprise managers and technicians enhance their understanding and application capabilities of AI; grant enterprises in the western region more favorable AI R&D subsidies and tax reductions to narrow regional disparities.

Summary of Revisions:

Please refer to the sections highlighted in red in the manuscript for your review.

Reviewer 2 Comments:

Comment 1:In 2.2.3 section, the author elaborates on the impact of AI on the export technological complexity of manufacturing enterprises by enhancing innovation capability, and analyzes the influence of AI on the innovation from three aspects. It is suggested to organize these three points into paragraph and use conjunctions such as "firstly", "secondly", and "thirdly".

Revision Explanation:

Thank you for your suggestion. In the revised manuscript, the three aspects regarding AI's influence on innovation capability have been reorganized into a coherent paragraph using the conjunctions "firstly," "secondly," and "thirdly" as recommended.

Summary of Revisions:

Please refer to the sections highlighted in red in the manuscript for your review.

Comment 2:In all regression result tables, the significance of the data is not indicated by an asterisk (*). The capitalization of "yes" and "Yes" in "Table 4-1 Baseline regression result" is not consistent, and there is a lack of relevant explanation for significance identification in the Note.

Revision Explanation:

Your point regarding the table notations is crucial. All regression tables have been updated to include asterisks indicating statistical significance, the notation for "Yes" has been standardized, and a clear explanation of significance levels has been added to the notes.

Summary of Revisions:

Please refer to the sections highlighted in red in the manuscript for your review.

Comment 3:The regression results of the mediation mechanism tests in "Table 4-7" and "Table 4-8" did not control industry and year fixed effects.

Revision Explanation:

Regarding the control variables in the mechanism test tables, we have carefully reviewed and re-ran the regression analyses. The models in Tables 4-7 and 4-8 now include controls for both industry and year fixed effects.

Summary of Revisions:

Please refer to the sections highlighted in red in the manuscript for your review.

Comment 4:In“4. Empirical Analysis”section, it is suggested to place "4.3.4 Mechanism Testing" after "4.1 Regression Results".

Revision Explanation:

Following your feedback on the paper's structure, we have moved the "4.3.4 Mechanism Testing" section to follow "4.1 Regression Results" to improve the logical flow of the empirical analysis.

Summary of Revisions:

Please refer to the sections highlighted in red in the manuscript for your review.

Comment 5:Pay attention to standardizing the article format, indent the first line of all paragraphs, and adjust the font of the "5. Discussion" section.

Revision Explanation:

Thank you for your attention to the formatting details. The first line of all paragraphs has been properly indented, and the font in the "5. Discussion" section has been adjusted to conform to the required style guidelines.

Summary of Revisions:

Please refer to the sections highlighted in red in the manuscript for your review.

We tried our best to improve the manuscript and made some changes. These changes will not influence the content and framework of the paper. Once again, thank you very much for your comments and suggestions.

Thank you once again to the esteemed reviewers for your valuable comments!

We wish you all the very best of health and every success!

Yours sincerely,

Mengtian Wang

Attachment Submitted filename: Response.docx

# Editor report: Decision Letter 4

**DOI:** 10.1371/journal.pone.0351061.r009

**Authors:** Van Thanh Tien Nguyen, Van Thanh Tien Nguyen, Van Thanh Tien Nguyen, Van Thanh Tien Nguyen, Van Thanh Tien Nguyen

23 May 2026

Research on the Impact of Artificial Intelligence on the Export Technological Complexity of Chinese Manufacturing Enterprises: An Analysis Based on Mediating Effects

PONE-D-24-35896R4

Dear Dr. Li,

We’re pleased to inform you that your manuscript has been judged scientifically suitable for publication and will be formally accepted for publication once it meets all outstanding technical requirements.

Within one week, you’ll receive an e-mail detailing the required amendments. When these have been addressed, you’ll receive a formal acceptance letter and your manuscript will be scheduled for publication.

An invoice will be generated when your article is formally accepted. Please note, if your institution has a publishing partnership with PLOS and your article meets the relevant criteria, all or part of your publication costs will be covered. Please make sure your user information is up-to-date by logging into Editorial Manager at Editorial Manager® and clicking the ‘Update My Information' link at the top of the page. For questions related to billing, please contact billing support.

If your institution or institutions have a press office, please notify them about your upcoming paper to help maximize its impact. If they’ll be preparing press materials, please inform our press team as soon as possible -- no later than 48 hours after receiving the formal acceptance. Your manuscript will remain under strict press embargo until 2 pm Eastern Time on the date of publication. For more information, please contact onepress@plos.org.

Kind regards,

Van Thanh Tien Nguyen, Ph.D.

Academic Editor

PLOS One

Additional Editor Comments (optional):

Reviewers' comments:

# Editor report: Acceptance letter

**DOI:** 10.1371/journal.pone.0351061.r010

**Authors:** Van Thanh Tien Nguyen, Van Thanh Tien Nguyen, Van Thanh Tien Nguyen, Van Thanh Tien Nguyen, Van Thanh Tien Nguyen

PONE-D-24-35896R4

PLOS One

Dear Dr. Li,

I'm pleased to inform you that your manuscript has been deemed suitable for publication in PLOS One. Congratulations! Your manuscript is now being handed over to our production team.

At this stage, our production department will prepare your paper for publication. This includes ensuring the following:

* All references, tables, and figures are properly cited

* All relevant supporting information is included in the manuscript submission,

* There are no issues that prevent the paper from being properly typeset

You will receive further instructions from the production team, including instructions on how to review your proof when it is ready. Please keep in mind that we are working through a large volume of accepted articles, so please give us a few days to review your paper and let you know the next and final steps.

Lastly, if your institution or institutions have a press office, please let them know about your upcoming paper now to help maximize its impact. If they'll be preparing press materials, please inform our press team within the next 48 hours. Your manuscript will remain under strict press embargo until 2 pm Eastern Time on the date of publication. For more information, please contact onepress@plos.org.

You will receive an invoice from PLOS for your publication fee after your manuscript has reached the completed accept phase. If you receive an email requesting payment before acceptance or for any other service, this may be a phishing scheme. Learn how to identify phishing emails and protect your accounts at https://explore.plos.org/phishing.

If we can help with anything else, please email us at customercare@plos.org.

Thank you for submitting your work to PLOS ONE and supporting open access.

Kind regards,

PLOS ONE Editorial Office Staff

on behalf of

Asst. Prof. Van Thanh Tien Nguyen

Academic Editor

PLOS One
